# Exploring the Pleiotropic Genes and Therapeutic Targets Associated with Heart Failure and Chronic Kidney Disease by Integrating metaCCA and SGLT2 Inhibitors' Target Prediction

**DOI:** 10.1155/2021/4229194

**Published:** 2021-09-08

**Authors:** Huanqiang Li, Ziling Mai, Sijia Yu, Bo Wang, Wenguang Lai, Guanzhong Chen, Chunyun Zhou, Jin Liu, Yongquan Yang, Shiqun Chen, Yong Liu, Jiyan Chen

**Affiliations:** ^1^Department of Cardiology, Guangdong Provincial Key Laboratory of Coronary Heart Disease Prevention, Guangdong Cardiovascular Institute, Guangdong Provincial People's Hospital, Guangdong Academy of Medical Sciences, Guangzhou 510080, China; ^2^Guangdong Provincial People's Hospital, School of Biology and Biological Engineering, South China University of Technology, Guangzhou 510080, China; ^3^The Second School of Clinical Medicine, Southern Medical University, Guangzhou, 510515 Guangdong, China; ^4^Guangdong Provincial People's Hospital, School of Medicine, South China University of Technology, Guangzhou 510515, China

## Abstract

**Background:**

Previous studies have shown that heart failure (HF) and chronic kidney disease (CKD) have common genetic mechanisms, overlapping pathophysiological pathways, and therapeutic drug—sodium-glucose cotransporter 2 (SGLT2) inhibitors.

**Methods:**

The genetic pleiotropy metaCCA method was applied on summary statistics data from two independent meta-analyses of GWAS comprising more than 1 million people to identify shared variants and pleiotropic effects between HF and CKD. Targets of SGLT2 inhibitors were predicted by SwissTargetPrediction and DrugBank databases. To refine all genes, we performed using versatile gene-based association study 2 (VEGAS2) and transcriptome-wide association studies (TWAS) for HF and CKD, respectively. Gene enrichment and KEGG pathway analyses were used to explore the potential functional significance of the identified genes and targets.

**Results:**

After metaCCA analysis, 4,624 SNPs and 1,745 genes were identified to be potentially pleiotropic in the univariate and multivariate SNP-multivariate phenotype analyses, respectively. 21 common genes were detected in both metaCCA and SGLT2 inhibitors' target prediction. In addition, 169 putative pleiotropic genes were identified, which met the significance threshold both in metaCCA analysis and in the VEGAS2 or TWAS analysis for at least one disease.

**Conclusion:**

We identified novel variants associated with HF and CKD using effectively incorporating information from different GWAS datasets. Our analysis may provide new insights into HF and CKD therapeutic approaches based on the pleiotropic genes, common targets, and mechanisms by integrating the metaCCA method, TWAS and VEGAS2 analyses, and target prediction of SGLT2 inhibitors.

## 1. Introduction

It is estimated that more than 23 million people worldwide are currently affected by heart failure (HF) [1], which dramatically increases the burden of finance and medical care [2, 3]. Although almost any disease that affects myocardial function can aggravate the development of HF, the disease most related to HF is chronic kidney disease (CKD). The relation of HF and CKD is tight. On the one hand, as a major complication of HF, CKD accelerates the overall progression of HF, resulting in higher mortality [4, 5]. On the other hand, HF is a leading cause of morbidity and mortality in patients with chronic kidney disease (CKD) and almost 30% of CKD patients are comorbid with HF [5, 6].

It is reported that the estimated heritability ratio of CKD is about 30-50% [7], while HF had an estimated heritability of 26-34% [8], indicating HF and CKD have solid genetic components. Genes including FTO and PKR1 have been jointly investigated in HF and CKD [9, 10]. Currently, dapagliflozin, one of the sodium-glucose cotransporter 2 (SGLT2) inhibitors, has been approved in the United States to treat adult patients with CKD regardless of diabetes status [11]. Data to date suggest SGLT2 inhibitors appear to moderately reduce the risk of the decrease of estimated glomerular filtration rate (eGFR), progression to end-stage renal disease (ESRD), cardiovascular (CV) death, and hospitalization for heart failure beyond simply reducing plasma glucose level [12–17]. Apparently, SGLT2 inhibitors are effective in both HF and CKD with some potential common therapeutic targets. However, the specific genetic and molecular mechanisms, as well as the common therapeutic targets shared by HF and CKD, are far from understanding.

As far as we know, the shared mechanism and risk molecular of HF and CKD have been discussed at proteomic and epigenetic levels [18, 19]. However, current studies have not systematically revealed the shared risk genes of HF and CKD. As a systematic and standard univariate detection method, genome-wide association study (GWAS) mainly provides an available way to identify diseases by testing one single-nucleotide polymorphism (SNP) and one quantitative phenotype at a time [20]. Several large-scale GWAS have been carried out in the past few years to find genetic variations of HF and CKD, respectively [21, 22]. However, these GWAS have achieved limited success in identifying genetic structure when it comes to complex traits and diseases such as HF and CKD. Even the test sample is large enough, GWAS can still not account for the entire relationship between genes and phenotypes, as most gene sites have little effect on traits [23]. Another limitation of GWAS is that the identified SNP can only explain part of the narrow heritability [24]. As a result, though GWAS can successfully identify disease-sensitive points, most genetic components of phenotypic variation remain unexplained in common diseases and common variations.

The canonical correlation analysis that uses multivariate statistics to represent genotypic and phenotypic variables based on published univariate GWAS statistics may be suitable for overcoming GWAS limitations [25]. Genotype-phenotypic associations in the genetic variation of most complex diseases can only be found when several variables are tested together. Thus, we can test the correlation between multiple SNP and multiple phenotypes. This method has been applied to identify 67 pleiotropic genes associated with seven autoimmune/autoinflammatory diseases [26]. At the same time, in the cardiovascular field, the metaCCA method has also been used to reveal the genetic association among CAD, obesity, and T2DM [27]. However, these studies only integrate genome data, resulting in only 67 and 22 putative pleiotropic genes obtained separately and failed to reveal the potential shared therapeutic targets of diseases.

In this study, we verified the results of metaCCA by combining the multiomics dataset rather than just using genome data to identify 169 disease-associated pleiotropic genes. Potential therapeutic targets of SGLT2 inhibitors shared by HF and CKD were also detected. In addition, we conduct functional enrichment and PPI network to explore the genes and therapeutic targets we identified.

## 2. Method

### 2.1. GWAS Datasets

The GWAS dataset for heart failure was adopted from a meta-analysis containing 26 studies that comprise 47,309 cases and 930,014 controls of European ancestry. Its summary statistics of 8,021,999 imputed SNPs were downloaded from http://www.broadcvdi.org/. Cases refer to participants with a clinical diagnosis of HF of any etiology without inclusion criteria based on LV ejection fraction; controls were participants without HF [21]. The adopted GWAS meta-analysis of CKD was composed of 60 studies with a total sample size of 689,383 including 64,164 CKD cases and 625,219 controls of European ancestry, and 9,585,589 SNPs for summary statistics. Patients with an eGFR below 60 ml min^−1^ per 1.73 m^2^ were defined as CKD regardless of etiology [22]. And its summary statistics were downloaded from https://ckdgen.imbi.uni-freiburg.de. We referred to every original study of these two GWAS meta-analyses to ensure all the samples in the GWAS datasets came from different populations with European ancestry. Additionally, the summary statistics have undertaken double check of genomic control separately at the individual study level and meta-analysis. Please refer to the corresponding consortium publications for more detailed information on the sample ascertainment and stringent quality control procedures.

### 2.2. Data Processing

After downloading summary statistics, we took several steps to process data for implementation of the metaCCA method. First, we combined the summary statistics to identify common SNPs between studies of HF and CKD. Then 7,754,982 overlapping SNPs of HF and CKD have completed the gene annotation for the two GWAS according to the 1000 Genomes dataset using PLINK1.9 (http://www.cog-genomics.org/static/bin/plink/glist-hg19). Only the SNPs that can be annotated were reserved for the following procedures. Second, we conducted a linkage disequilibrium (LD) to remove SNPs with large pairwise correlations based on the SNP pruning method. The SNP pruning method was proceeded by a window size of 50 SNPs and step size of 5 SNPs, which means LD was calculated between each pair of SNPs to remove SNPs in high LD; each sliding window of 5 SNPs moved forward and the process repeated until there were no pairs of SNPs with high LD [28]. SNPs with smaller minor allele frequency (MAF) for pairs with *r*^2^ > 0.2 were also removed [29]. All datasets were expurgated using the 1000 Genomes genotypes of CEU as a reference panel. After gene annotation and SNP pruning, there remained 440,440 SNPs which we performed the metaCCA analysis. For each of these remaining SNPs, we obtained the regression coefficient *β* and its standard error. The regression coefficient *β* was normalized before conducting the metaCCA analysis. Standardization was achieved according to the following equation:

(1) $$\begin{array}{c} \beta_{gp}^{\text{STD}}=\frac{1}{\sqrt{n{\text{SE}}_{gp}}}\times \beta_{gp}, \end{array}$$

where SE_*gp*_ is the standard error of *β*_*gp*_. Both SE*_gp_* and *β_gp_* are given by the original GWAS result; *g* is the number of genotypic variables, *p* is the number of phenotypic variables, and *n* is the sample number of each disease.

### 2.3. metaCCA Analysis

To identify the potential pleiotropic genes, we applied the metaCCA method. The principle and algorithm of metaCCA were consistent with the description of the paper of Cichonska et al. [25]. In short, metaCCA is a multivariate meta-analysis method which is an extension of the method of CCA and requires a cross-covariance matrix between all genotypic and phenotypic variables (∑*XY*), a genotypic correlation structure among SNPs (∑^∧^*XX*), and a phenotypic correlation structure among traits (∑^∧^*YY*) [25]. ∑*XY* is constructed as the normalized regression coefficient *β*_*gp*_:

(2) $$\begin{array}{c} \sum XY=\frac{X^{T}Y}{N-1}=\left(\begin{array}{ccc} \beta_{11} & \cdots & \beta_{1p}\\ \vdots & \ddots & \vdots \\ \beta_{g1} & \cdots & \beta_{gp} \end{array}\right). \end{array}$$

In the formula, *g* and *p* are the numbers of genotypic and phenotypic variables, respectively.

In our study, ∑^∧^*XX* was calculated using the 1000 Genomes reference for Europeans (phase 3) as a reference representing the study population. Furthermore, the phenotypic correlation structure (∑^∧^*YY*) was computed based on ∑*XY*. Each entry of ∑^∧^*YY* corresponded to a Pearson correlation coefficient between the vector of *β* estimates from *p* phenotypic variables across *g* genetic variants. For the sample size varies between these two studies, we set the smallest sample size among the included traits as *N*, which is the most conservative and commonly used approach to address this issue. After calculation, the full covariance matrix (∑) can be obtained:

(3) ∑=∑^XX∑XY∑XYT ∑^YY.

The full covariance matrix was plugged into the CCA framework to produce the final genotype-phenotype association result [25]. The correlation between genotype and phenotype is called the canonical correlation *r* [30]. In this study, we conducted two types of multivariate analysis: univariate SNP-multivariate phenotype association analysis for the SNP level and multivariate SNP-multivariate phenotype association analysis for the gene level. The result was the canonical correlation of a gene with HF and CKD. Then, Bonferroni corrected *P* value < 0.01 was used as the threshold for significance. If the *P* value of the canonical correlation *r* of any SNP was less than 2.77 × 10^−8^ ( = 0.01/440440), it was deemed significantly associated with HF and CKD. Similarly, genes with a canonical correlation *P* value smaller than 6.54 × 10^−7^ ( = 0.01/15302) were significantly associated with HF and CKD.

### 2.4. Prediction of SGLT2 Inhibitors' Related Targets

The chemical structures of four SGLT2 inhibitors, namely, canagliflozin, dapagliflozin, empagliflozin, and ertugliflozin, were obtained using PubChem (https://pubchem.ncbi.nlm.nih.gov/), which is an open chemistry database with 96,502,248 compositions of which 3,151,393 have been tested. We adopted SwissTarget Prediction (http://www.swisstargetprediction.ch/), a tool for target prediction according to dimensional and 3-dimensional similarity measures with known ligands, to select and to predict potential targets for four SGLT2 inhibitors by putting their chemical structures into this platform [31]. Additionally, SGLT2 inhibitors' related genes were also collected from DrugBank (https://www.drugbank.ca/), a unique bioinformatics and chemical informatics database containing 11,628 drugs and related chemical information, drug targets, protein data, and so on [32]. With further correction and transformation by retrieving Universal Protein Resource (UniProt, http://www.uniprot.org/), all the SGLT2 inhibitors' related genes were normalized into consistent symbols for subsequent analysis. According to the intersection of genes identified by metaCCA and SGLT2 inhibitors' related targets, we get the Venn diagram on the website (https://bioinfogp.cnb.csic.es/tools/venny/).

### 2.5. Gene-Based Analysis

To make the identified genes by metaCCA more credible, we performed gene-based association analysis by using the versatile gene-based association study 2 (VEGAS2) method (performed at https://vegas2.qimrberghofer.edu.au/). This method is aimed at determining the association between each SNP and each trait using original GWAS summary statistics individually [33]. All SNPs in each gene were analyzed using the 1000 Genomes European reference genotypes. Our study identified significant genes that achieved threshold in the VEGAS2 analysis using 0.01 as the *P* value threshold.

### 2.6. Transcriptome-Wide Association Studies

Transcriptome-wide association studies (TWAS) of HF and CKD were performed using FUSION software. FUSION can compute gene expression weights for different tissues by individual's genotype and gene expression data. In the current study, gene expression weights of the whole blood panel were adopted from the FUSION website (https://gusevlab.org/projects/fusion/), which is attached to the GTEx database. Genes with corrected *P* values < 0.05 were identified as significant risk genes. For more information of FUSION parameters, please refer to the previous article [34].

### 2.7. Functional GO and KEGG Analyses and PPI Network Conduction

**Figure 1:**
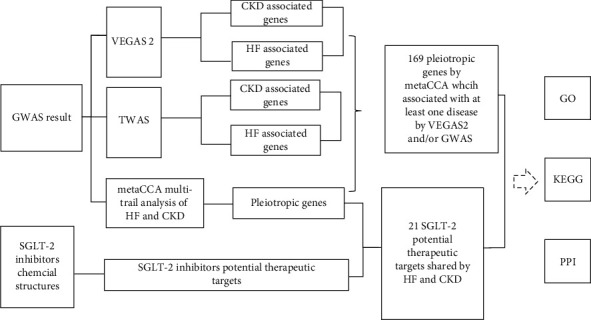
The workflow of the study.

These putative pleiotropic genes and potential pleiotropic therapeutic targets identified by metaCCA and SGLT2 target prediction were then subjected to GO and KEGG analyses. The Database for Annotation, Visualization, and Integrated Discovery (DAVID) bioinformatics tool was used to evaluate these analyses. A GO term or KEGG pathway with an adjusted *P* value < 0.05 was considered statistically significant [35]. Visualization of GO analysis was conducted with R package “ggplot2.” In addition, we used STRING v10 from https://string-db.org/ to analyze the PPI network [36]. The workflow of the whole study is shown in Figure 1.

## 3. Result

### 3.1. Pleiotropic SNPs and Genes Identified by metaCCA

**Figure 2:**
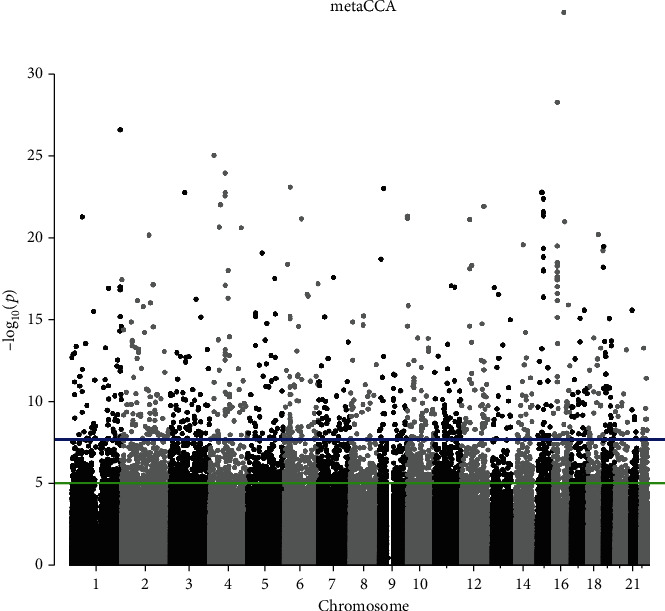
Manhattan plot of −log_10_(*P*_metaCCA_) values for univariate HF and CKD analyses. The blue line marks the −log_10_(*P*_metaCCA_) corresponding to *P* < 1.13 × 10^−7^. The green line marks the −log_10_(*P*_metaCCA_) corresponding to *P* < 3.16 × 10^−7^.

After gene annotation and SNP pruning, 440,440 SNPs located in 23,579 gene regions were inputted in the metaCCA analysis. Totally, 4,624 SNPs reached the Bonferroni corrected threshold (*P* < 2.77 × 10^−8^), and the canonical correlation *r* between each SNP and phenotype ranged from 0.005 to 0.013. The Manhattan plot in Figure 2 presents the results. For the multivariate SNP-multivariate phenotype analysis, 1,745 genes with a significance threshold of *P* value (*P* < 6.54 × 10^−7^) were identified as the potential pleiotropic genes. The canonical correlation *r* between genotype and phenotype ranged from 0.008 to 0.069.

### 3.2. Potential Pleiotropic Therapeutic Targets Identified by metaCCA and SGLT2 Target Prediction

**Figure 3:**
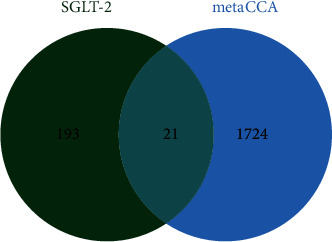
Venn diagram of multi-SNP-metaCCA and SGLT2 target prediction.

After importing four SGLT2 inhibitors' structures obtained from PubChem into the SwissTargetPrediction database, respectively, for target matching and prediction, we screened the 400 targets (Supplementary Table 1). We also adopted the DrugBank database to find 37 targets on the four SGLT2 inhibitors, with 11 targets in dapagliflozin, 10 targets in empagliflozin, 8 targets in canagliflozin, and 8 targets in ertugliflozin (Supplementary Table 2). By integrating and eliminating duplicate targets in the two databases, a total of 214 targets with potential effects of SGLT2 inhibitors were obtained. An intersection of genes identified by metaCCA and SGLT2 inhibitors' related targets was obtained from the online Venn diagram drawing website (http://jvenn.toulouse.inra.fr/app/example.html). We finally identified 21 potential therapeutic targets for SGLT2 inhibitors in both heart and kidney failures, including SLC5A1, EP300, and GRIK1, which were also considered to be pleiotropic genes. The results are presented by the Venn plot in Figure 3. For detailed information of potential therapeutic targets, please refer to Table 1.

### 3.3. Pleiotropic Genes Identified by metaCCA and VEGAS2 Analyses and TWAS Analysis

**Figure 4:**
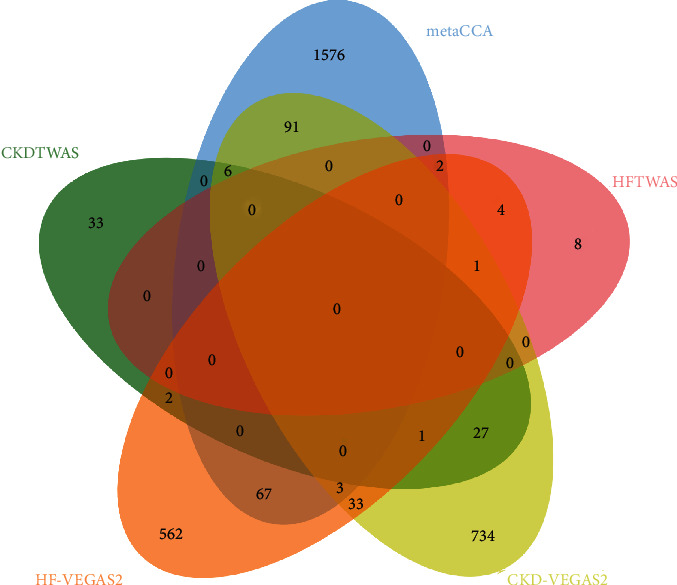
Venn diagram of multi-SNP-metaCCA and HF- and CKD-VEGAS2 and HF- and CKD-TWAS analyses.

To refine and validate our multivariate SNP-multivariate phenotype analysis, we then integrated gene-based analysis using the VEGAS2 and TWAS analyses. For VEGAS2 analysis, 675 genes of HF and 897 genes of CKD reached the threshold of significance. As for TWAS analysis, we identified 15 genes associated with HF and 69 genes associated with CKD. After intercepting the result of VEGAS2 analysis and TWAS analysis with 1,745 pleiotropic genes identified by metaCCA, we determined 169 putative pleiotropic risk genes associated with at least one disease in the VEGAS2 analysis or TWAS analysis (Figure 4). Among the 169 putative pleiotropic genes, some genes have been detected in the original GWAS meta-analysis, such as KCNQ1 (*P*_metaCCA_ = 3.01 × 10^−19^, *P*_CKD−meta_ = 9.02 × 10^−9^) for CKD and ATXN2 (*P*_metaCCA_ = 1.87 × 10^−9^, *P*_HF−meta_ = 4.90 × 10^−8^) and FTO (*P*_metaCCA_ = 7.54 × 10^−147^, *P*_HF−meta_ = 1.21 × 10^−8^) for HF, indicating that metaCCA have a more statistic power. In comparison, 100 genes were determined as novel putative pleiotropic genes because these genes have never been reported to be associated with HF and CKD (Supplementary Table 3).

Three common genes, FTO, SLC39A8, and CRTC1, were identified in metaCCA analysis and two diseases in VEGAS2 analysis. Two common genes, POM121C and NPC1, were identified in both VEGAS2 and TWAS of HF and metaCCA analyses, while six common genes, including ALMS1P, GAB2, PAX8, GGNBP2, L3MBTL3, and GATM, identified in both VEGAS2 and TWAS of CKD and metaCCA analyses. Of these common genes, FTO and SLC39A8 have been reported to be associated with HF and CKD. PAX8 has been reported to be associated with both HF and CKD. GATM was previously reported to be associated with CKD, while other genes were determined as novel pleiotropic genes. These common genes are summarized in Table 2.

### 3.4. Functional Enrichment Analysis and PPI Network Conduction

**Figure 5:**
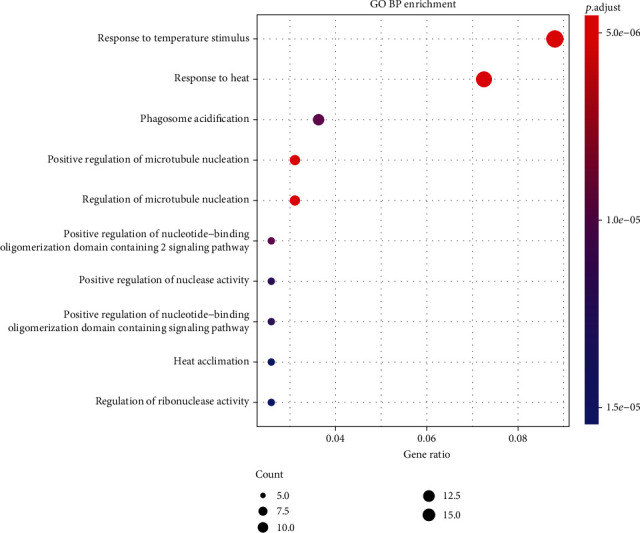
Biological process dot plot of the top 10 GO terms of 169 putative pleiotropic genes and 21 potential pleiotropic therapeutic targets for SGLT2 inhibitors.

**Figure 6:**
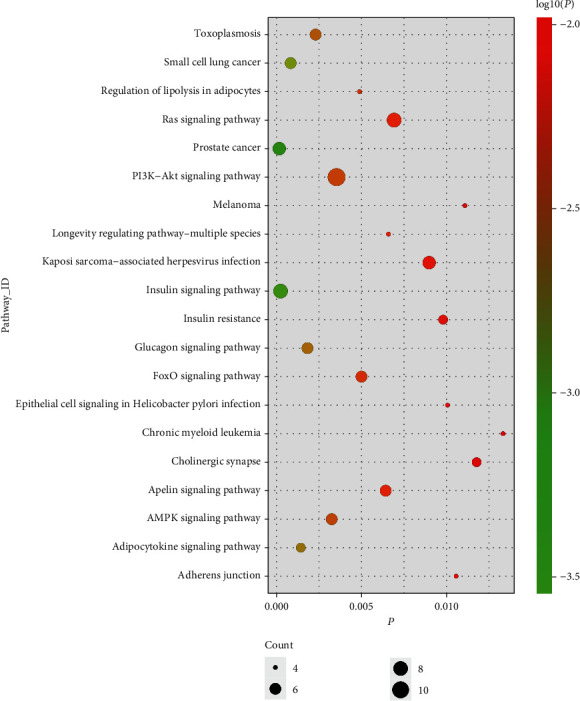
KEGG dot plot of the top 20 pathways enrichment of 169 putative pleiotropic genes and 21 potential pleiotropic therapeutic targets for SGLT2 inhibitors.

**Figure 7:**
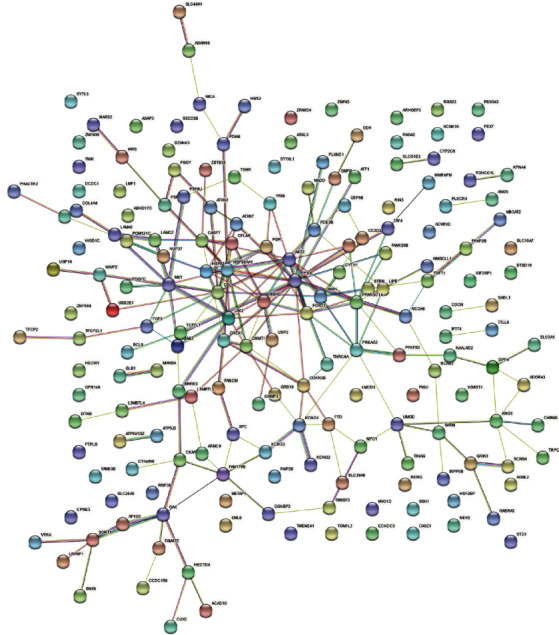
PPI network of 169 putative pleiotropic genes and 21 potential pleiotropic therapeutic targets for SGLT2 inhibitors.

To explore the potential functional significance of the identified genes, we conducted GO enrichment analysis and KEGG analysis to reveal the biological functions and involved pathways of these pleiotropic genes. What the top five GO biological process terms identified from 169 pleiotropic genes and 21 potential pleiotropic therapeutic targets of SGLT2 inhibitors associated with HF and CKD were response to temperature stimulus (GO:0009266), positive regulation of microtubule nucleation (GO:0090063), regulation of microtubule nucleation (GO:0010968), response to heat (GO:0009408), and phagosome acidification (GO:0090383). Biological process dot plot of the top 10 GO terms of 169 putative pleiotropic genes is presented in Figure 5. And for KEGG analysis, the top five descriptions were prostate cancer (hsa05215), the insulin signaling pathway (hsa04910), small-cell lung cancer (hsa05222), the adipocytokine signaling pathway (hsa04920), and the glucagon signaling pathway (hsa04922). Other pathways confirmed to be associated with HF and CKD were also identified, such as the AMPK signaling pathway (hsa04152) and the apelin signaling pathway (hsa04371). The top twenty pathways are drawn in Figure 6. We also conducted a PPI network to determine the interaction of the proteins encoded by these pleiotropic genes, consisting of 183 nodes and 202 edges. The average node degree was 2.21 (Figure 7).

## 4. Discussion

In this study, we used two independent GWAS meta-analyses with available summary statistics of HF and CKD to perform the multivariate pleiotropic mapping. Potential therapeutic targets of SGLT2 inhibitors shared by HF and CKD were determined by the intersection of genes in metaCCA and SGLT2 inhibitors' related target analysis. Furthermore, genes determined by metaCCA have then obtained a verification using VEGAS2 analysis and TWAS analysis. We finally identified a total of 21 potential therapeutic targets and 169 putative pleiotropic genes. Moreover, we performed the functional enrichment analysis based on these results. In KEGG analysis based on putative pleiotropic genes and potential therapeutic targets, we identified several pleiotropic pathways that have been confirmed to be shared by HF and CKD, which may also play an important role in the SGLT2 inhibitors pharmacological mechanism. The result of functional enrichment provided us with a better understanding of the potential common biological pathogenesis of HF and CKD.

Seven out of the twenty-one therapeutic targets have been reported to be associated with both HF and CKD. In the remaining genes, only GRIK1, FDFT1, and PFKFB3 were first reported to be therapeutic targets of HF and CKD. Previous studies have demonstrated therapeutic mechanisms for some potential pleiotropic therapeutic targets of HF and CKD identified by metaCCA and SGLT2 target prediction. For example, sodium-glucose cotransporter SGLT1 (also known as SLC5A1) accounts for most of the dietary glucose uptake [37]. SLC5A1 inhibition can protect against myocardial infarction-induced ventricular remodeling and heart failure in mice by replenishing ATP stores in ischemic cardiac tissues by enhancing glucose availability [38]. In an epigenome-wide association study of DNA methylation associated with kidney function, Chu et al. found that the regions containing the 243 eGFR-associated CpGs are significantly enriched for transcription factor binding sites of EP300 [39]. And Witt et al. found a linear correlation between EP300 antisense RNA1 and left ventricular ejection fractions (LVEF) [40]. However, since EP300 has yet been fully studied in HF, it may be the next therapeutic target worth exploring for SGLT2 inhibitors.

Among 169 putative pleiotropic genes, 100 genes are identified as novel, while 26 genes have been reported to be critical to HF or CKD etiology. For example, FTO is a confirmed pleiotropic gene, which encodes an N6-methyladenosine (m6A) RNA demethylase [41]. In addition, the A allele of the FTO rs708259 polymorphism may be a relevant genetic risk factor of CKD because it has been proved to be an independent predictor of all-cause mortality in patients with various severity of CKD [42]. As for SLC39A8, this gene encodes the ZIP8 metal cation transporter in all vertebrates. Olgar et al. found that the expressions of ZIP14 and ZIP8 were significantly increased, while the level of ZIP8 decreased in HF [43]. In CKD, iron deposition is associated with increased intensity of iron importers (ZIP14 and ZIP8), indicating that the result from altered molecular iron handling may contribute to renal injury [44]. Compared to previous studies, we integrated multitrait and multiomic analyses to determine putative pleiotropic genes rather than validate with only genome data. By taking gene expression of specific tissues into account, several genes with relative insignificance of metaCCA are more worthy of exploration. PAX8 and GATM are such two genes which have been reported to be associated with CKD overlapped in metaCCA, CKD-TWAS, and CKD-VEGAS2 analyses needed to be further explored the association with HF [45, 46].

Several confirmed pleiotropic pathways shared by HF and CKD were also detected by enrichment analysis, including the insulin signaling pathway, the AMPK signaling pathway, and the apelin signaling pathway. These pathways may also play an important role in the SGLT2 inhibitors' pharmacological mechanism. Activation of the proximal insulin signaling pathways within cardiomyocytes increases when HF may contribute to adverse left ventricular remodeling and mitochondrial dysfunction [47]. Insulin resistance and acquired defects in the insulin-receptor signaling pathway are common in CKD patients due to their complex metabolic abnormalities [48].

Adenosine 5′-monophosphate- (AMP-) activated protein kinase (AMPK) is a critical molecule in the regulation of bioenergy metabolism [49]. It is the core of researches on diabetes and other metabolism-related diseases such as HF and CKD [50, 51]. Dapagliflozin can increase the P-AMPK/total-AMPK ratio in the type 2 diabetic mice, attenuating the activation of the inflammasome, fibrosis, and deterioration of left ventricular ejection fractions [52]. Since dapagliflozin has been proved by FAD for the therapy of CKD patients, the function of the AMPK signaling pathway between SGLT2 inhibitors and CKD needs further investigation. The apelin signaling pathway is common in HF and CKD [53, 54]. Apelin abnormalities enhance the progressive impairment of myocardial contractility and systolic dysfunction, and loss of apelin contributes to HF in response to pressure overload [55]. Apelin/APJ system has been reported to alleviate CKD by inhibiting vascular calcification [56].

Two original GWAS included cohorts with either a case-control or population-based study design regardless of the etiologies of HF or CKD. Although HF and CKD are complex clinical syndromes with various etiologies, the result of this study cannot be specific to any type of HF or CKD. However, the strong correlation between these pathways with HF and CKD proves the reliability and popularity of our results.

To our knowledge, this study is the first study using the metaCCA method to identify shared risk genes and therapeutic targets of SGLT2 inhibitors associated with HF and CKD by effectively incorporating information from different GWAS datasets. Learning pleiotropic genes and their effects is essential. Compared to the animal experiments or one-trait GWAS, there are some advantages of our study. Firstly, the statistical power of the present study is higher than previous studies by integrating two large-scale GWAS summary statistics, which provided an increase in effective sample size up to 1 million. Secondly, the metaCCA method used published GWAS data, which does not require sequencing at the individual level, so it is a cost-effective method. Finally, with the help of the principle of the multitrait and multiomics, our study not only considered the internal correlation between genotypes but also the correlation between HF and CKD phenotypes [57]. Using the correlation between multivariate phenotypic variables, the strategy of extracting similar principal component factors from multivariate phenotypic variables for correlation analysis is an effective method to identify rare variations in complex diseases [58]. Thus, this study not only verified pleiotropic genes with higher statistic power existing in HF and CKD but also detected novel pleiotropic genes.

In the meantime, there are some limitations of this study. First of all, metaCCA mainly relies on statistical analysis, in which false positive results may occur. However, this may be addressed through the utilization of VEGAS2 analysis for validation of pleiotropic genes identified by metaCCA. It is still easy to have a particular SNP strongly correlated with multiple phenotypes making the gene or pathway in which the SNP is located meaningful [59]. Secondly, metaCCA integrates different GWAS summary statistics into one, so the processing of factors may be different. GWAS summary statistics without the genotype data at the individual level limit us to determine the proportions of variability explained by the genes [25]. Thirdly, those novel therapeutic targets of SGLT2 were predicated based on online tools, which require further experimental evidence. Lastly, other gene-level statistical methods or multivariate statistical methods or further experimental studies are needed to screen the results of metaCCA and confirm the novel findings.

Our study performed a multivariate analysis by integrating VEGAS2 and TWAS to detect 169 pleiotropic genes. Moreover, we detected 21 therapeutic targets of SGLT2 that are associated with HF and CKD by integrating metaCCA and SGLT2 inhibitors' target prediction. In addition, we also illustrated the potential biological functions of these pleiotropic genes and therapeutic targets. Our results may provide novel insights into the shared genetic factors in the development of HF and CKD and common therapeutic targets of SGLT2 inhibitors shared by HF and CKD.

## Figures and Tables

**Table 1:** Potential therapeutic genes identified by metaCCA and SGLT2 target prediction.

Gene	metaCCA *P* value	Gene type—HF	Gene type—CKD
SLC5A1	1.0000*E* − 315	Confirmed	Confirmed
EP300	2.3025*E* − 109	Confirmed	Confirmed
GRIK1	1.86359*E* − 100	Novel	Novel
CYP2C9	3.54433*E* − 54	Confirmed	Confirmed
IARS	6.39725*E* − 53	Novel	Novel
ADORA3	4.84074*E* − 49	Confirmed	Confirmed
DPP4	4.7235*E* − 46	Confirmed	Confirmed
FDFT1	7.65889*E* − 41	Novel	Novel
CHEK1	2.26504*E* − 21	Confirmed	Novel
MANBA	4.53369*E* − 21	Novel	Confirmed
HSP90AA1	2.65344*E* − 20	Confirmed	Novel
MET	3.74765*E* − 20	Confirmed	Novel
CASP7	2.8386*E* − 17	Confirmed	Novel
GAK	1.09751*E* − 16	Novel	Novel
DNMT1	1.63864*E* − 13	Confirmed	Confirmed
SCN9A	5.92972*E* − 11	Confirmed	Novel
JAK2	8.10834*E* − 11	Confirmed	Confirmed
IKBKB	9.40369*E* − 11	Novel	Confirmed
SLCO1B3	5.69126*E* − 10	Novel	Confirmed
PFKFB3	8.00898*E* − 10	Confirmed	Novel
GLB1	1.61052*E* − 09	Novel	Novel

Confirmed: this gene was previously reported to be associated with disease. Novel: this gene had never been reported to be associated with HF or CKD.

**Table 2:** Common pleiotropic genes identified by TWAS, metaCCA, and VEGAS2.

Gene	chr	metaCCA·*P*	HF-VEGAS2·*P*	CKD-VEGAS2·*P*	HF-TWAS·*P*	CKD-TWAS·*P*	Gene type
POM121C	chr7	1.50*E* − 11	7.09*E* − 04	5.68*E* − 01	4.55*E* − 02	8.27*E* − 01	Novel
NPC1	chr18	8.76*E* − 270	9.00*E* − 06	5.10*E* − 02	9.87*E* − 01	9.86*E* − 01	Novel
GGNBP2	chr17	6.17*E* − 68	3.92*E* − 02	1.00*E* − 06	6.85*E* − 01	3.15*E* − 02	Novel
L3MBTL3	chr6	3.29*E* − 25	7.37*E* − 02	1.23*E* − 04	5.45*E* − 01	1.47*E* − 02	Novel
GATM	chr15	3.75*E* − 09	3.04*E* − 01	1.00*E* − 06	8.38*E* − 01	7.77*E* − 07	Potential
GAB2	chr11	6.41*E* − 10	8.56*E* − 01	1.80*E* − 05	—	4.61*E* − 02	Novel
ALMS1P	chr2	4.63*E* − 11	1.56*E* − 01	1.13*E* − 04	8.20*E* − 01	7.03*E* − 04	Novel
PAX8	chr2	5.59*E* − 19	6.58*E* − 01	3.80*E* − 05	8.98*E* − 01	2.48*E* − 02	Potential
FTO	chr16	7.55*E* − 147	8.80*E* − 05	9.80*E* − 04	9.12*E* − 01	2.68*E* − 01	Confirmed
SLC39A8	chr4	1.29*E* − 27	5.78*E* − 03	4.43*E* − 03	6.33*E* − 01	7.88*E* − 01	Confirmed
CRTC1	chr19	1.21*E* − 08	7.44*E* − 03	9.50*E* − 03	—	—	Novel

Confirmed: this gene was previously reported to be associated with two diseases. Potential: this gene had been reported to be associated with only one disease of HF or CKD. Novel: this gene had never been reported to be associated with HF or CKD.

## Data Availability

The GWAS summary statistics data used to support the findings of this study have been deposited in the http://www.broadcvdi.org/, https://ckdgen.imbi.uni-freiburg.de.

## Abbreviations

HF: Heart failure
CKD: Chronic kidney disease
SGLT2: Sodium-dependent glucose transporter 2
metaCCA: Summary statistics-based multivariate meta-analysis of genome-wide association studies using canonical correlation analysis
GWAS: Genome-wide association study
TWAS: Transcriptome-wide association study
SNP: Single-nucleotide polymorphism
GO: Gene Ontology
KEGG: Kyoto Encyclopedia of Genes and Genomes
PPI: Protein-protein interaction
m6A: N6-methyladenosine
AMPK: Adenosine 5′-monophosphate- (AMP-) activated protein kinase
eGFR: Estimated glomerular filtration rate
ESRD: End-stage renal disease
CV: Cardiovascular death
HHF: Hospitalization for heart failure.

## Supplementary Materials

Supplementary 1 Table S1: SwissTargetPrediction—four SGLT2 inhibitors.

Supplementary 2 Table S2: DrugBank database—four SGLT2 inhibitors.

Supplementary 3 Table S3: 169 putative pleiotropic genes.

Supplementary 4 Table S4: GO BP enrichment.

Supplementary 5 Table S5: potential genes KEGG.
