# Supplementary material for: Exploring the Pleiotropic Genes and Therapeutic Targets Associated with Heart Failure and Chronic Kidney Disease by Integrating metaCCA and SGLT2 Inhibitors' Target Prediction
Source: Biomed Res Int. 2021 Sep 8;2021:4229194. doi: 10.1155/2021/4229194 (PMC8443964; doi:10.1155/2021/4229194)
Supplement: Supplementary 1 — Table S1: SwissTargetPrediction—four SGLT2 inhibitors. [file 4229194.f1.docx]

| SGLT2 inhibitors | Target | Common name | Uniprot ID | ChEMBL ID | Target Class | Probability* |
| --- | --- | --- | --- | --- | --- | --- |
| Dapagliflozin | Sodium/myo-inositol cotransporter 2 | SLC5A11 | Q8WWX8 | CHEMBL1744524 | Electrochemical transporter | 1 |
| Dapagliflozin | Low affinity sodium-glucose cotransporter | SLC5A4 | Q9NY91 | CHEMBL1770047 | Electrochemical transporter | 1 |
| Dapagliflozin | Sodium/glucose cotransporter 2 | SLC5A2 | P31639 | CHEMBL3884 | Electrochemical transporter | 1 |
| Dapagliflozin | Sodium/glucose cotransporter 1 | SLC5A1 | P13866 | CHEMBL4979 | Electrochemical transporter | 1 |
| Dapagliflozin | Adenosine kinase | ADK | P55263 | CHEMBL3589 | Enzyme | 0.115737 |
| Dapagliflozin | Platelet-derived growth factor receptor beta | PDGFRB | P09619 | CHEMBL1913 | Kinase | 0.115737 |
| Dapagliflozin | Tyrosine-protein kinase LCK | LCK | P06239 | CHEMBL258 | Kinase | 0.115737 |
| Dapagliflozin | MAP kinase p38 alpha | MAPK14 | Q16539 | CHEMBL260 | Kinase | 0.115737 |
| Dapagliflozin | c-Jun N-terminal kinase 3 | MAPK10 | P53779 | CHEMBL2637 | Kinase | 0.115737 |
| Dapagliflozin | MAP kinase p38 beta | MAPK11 | Q15759 | CHEMBL3961 | Kinase | 0.115737 |
| Dapagliflozin | Ephrin type-A receptor 5 | EPHA5 | P54756 | CHEMBL3987 | Kinase | 0.115737 |
| Dapagliflozin | Serine/threonine-protein kinase GAK | GAK | O14976 | CHEMBL4355 | Kinase | 0.115737 |
| Dapagliflozin | Adenosine A2a receptor | ADORA2A | P29274 | CHEMBL251 | Family A G protein-coupled receptor | 0.115737 |
| Dapagliflozin | Adenosine A3 receptor | ADORA3 | P0DMS8 | CHEMBL256 | Family A G protein-coupled receptor | 0.115737 |
| Dapagliflozin | Dual specificity mitogen-activated protein kinase kinase 1 | MAP2K1 | Q02750 | CHEMBL3587 | Kinase | 0.115737 |
| Dapagliflozin | Equilibrative nucleoside transporter 1 | SLC29A1 | Q99808 | CHEMBL1997 | Electrochemical transporter | 0.115737 |
| Dapagliflozin | Beta-glucosidase | GBA2 | Q9HCG7 | CHEMBL3761 | Enzyme | 0.115737 |
| Dapagliflozin | Beta-glucocerebrosidase | GBA | P04062 | CHEMBL2179 | Enzyme | 0.115737 |
| Dapagliflozin | Sodium/nucleoside cotransporter 2 | SLC28A2 | O43868 | CHEMBL5780 | Electrochemical transporter | 0.115737 |
| Dapagliflozin | Heat shock cognate 71 kDa protein | HSPA8 | P11142 | CHEMBL1275223 | Other cytosolic protein | 0.115737 |
| Dapagliflozin | 78 kDa glucose-regulated protein | HSPA5 | P11021 | CHEMBL1781865 | Unclassified protein | 0.115737 |
| Dapagliflozin | Hexokinase type II | HK2 | P52789 | CHEMBL2640 | Enzyme | 0.115737 |
| Dapagliflozin | Hexokinase type I | HK1 | P19367 | CHEMBL2688 | Enzyme | 0.115737 |
| Dapagliflozin | Phosphodiesterase 5A | PDE5A | O76074 | CHEMBL1827 | Phosphodiesterase | 0.115737 |
| Dapagliflozin | Coagulation factor VII/tissue factor | F3 | P13726 | CHEMBL4081 | Surface antigen | 0.115737 |
| Dapagliflozin | Liver glycogen phosphorylase | PYGL | P06737 | CHEMBL2568 | Enzyme | 0.115737 |
| Dapagliflozin | Uridine phosphorylase 1 (by homology) | UPP1 | Q16831 | CHEMBL4811 | Enzyme | 0.115737 |
| Dapagliflozin | Adenosine A2b receptor | ADORA2B | P29275 | CHEMBL255 | Family A G protein-coupled receptor | 0.115737 |
| Dapagliflozin | Cathepsin L | CTSL | P07711 | CHEMBL3837 | Protease | 0.115737 |
| Dapagliflozin | Glutathione S-transferase Pi | GSTP1 | P09211 | CHEMBL3902 | Enzyme | 0.115737 |
| Dapagliflozin | Glutathione S-transferase Mu 2 | GSTM2 | P28161 | CHEMBL4589 | Enzyme | 0.115737 |
| Dapagliflozin | Glucose transporter (by homology) | SLC2A1 | P11166 | CHEMBL2535 | Electrochemical transporter | 0.115737 |
| Dapagliflozin | Muscle glycogen phosphorylase | PYGM | P11217 | CHEMBL3526 | Enzyme | 0.115737 |
| Dapagliflozin | GABA receptor alpha-5 subunit | GABRA5 | P31644 | CHEMBL5112 | Ligand-gated ion channel | 0.115737 |
| Dapagliflozin | Epidermal growth factor receptor erbB1 | EGFR | P00533 | CHEMBL203 | Kinase | 0.115737 |
| Dapagliflozin | Alkaline phosphatase, tissue-nonspecific isozyme | ALPL | P05186 | CHEMBL5979 | Enzyme | 0.115737 |
| Dapagliflozin | Glyceraldehyde-3-phosphate dehydrogenase liver | GAPDH | P04406 | CHEMBL2284 | Oxidoreductase | 0.115737 |
| Dapagliflozin | Matrix metalloproteinase 3 | MMP3 | P08254 | CHEMBL283 | Protease | 0.115737 |
| Dapagliflozin | Matrix metalloproteinase 1 | MMP1 | P03956 | CHEMBL332 | Protease | 0.115737 |
| Dapagliflozin | Serine/threonine-protein kinase Chk1 | CHEK1 | O14757 | CHEMBL4630 | Kinase | 0.115737 |
| Dapagliflozin | Bifunctional protein NCOAT | OGA | O60502 | CHEMBL5921 | Enzyme | 0.115737 |
| Dapagliflozin | Purinergic receptor P2Y12 (by homology) | P2RY12 | Q9H244 | CHEMBL2001 | Family A G protein-coupled receptor | 0.115737 |
| Dapagliflozin | Glutamate [NMDA] receptor PROTEIN | GRIN1 | Q05586 | CHEMBL2015 | Ligand-gated ion channel | 0.115737 |
| Dapagliflozin | Interleukin-1 receptor-associated kinase 4 | IRAK4 | Q9NWZ3 | CHEMBL3778 | Kinase | 0.115737 |
| Dapagliflozin | Mitogen-activated protein kinase kinase kinase 14 | MAP3K14 | Q99558 | CHEMBL5888 | Kinase | 0.115737 |
| Dapagliflozin | Tyrosine-protein kinase JAK2 | JAK2 | O60674 | CHEMBL2971 | Kinase | 0.115737 |
| Dapagliflozin | Carbonic anhydrase XIV | CA14 | Q9ULX7 | CHEMBL3510 | Lyase | 0.115737 |
| Dapagliflozin | Alpha-L-fucosidase I | FUCA1 | P04066 | CHEMBL4176 | Enzyme | 0.115737 |
| Dapagliflozin | Intercellular adhesion molecule-1 | ICAM1 | P05362 | CHEMBL3070 | Adhesion | 0.115737 |
| Dapagliflozin | Vascular cell adhesion protein 1 | VCAM1 | P19320 | CHEMBL3735 | Adhesion | 0.115737 |
| Dapagliflozin | Selectin E | SELE | P16581 | CHEMBL3890 | Adhesion | 0.115737 |
| Dapagliflozin | Vascular endothelial growth factor receptor 3 | FLT4 | P35916 | CHEMBL1955 | Kinase | 0.115737 |
| Dapagliflozin | Neprilysin | MME | P08473 | CHEMBL1944 | Protease | 0.115737 |
| Dapagliflozin | Glutamate receptor ionotropic kainate 1 | GRIK1 | P39086 | CHEMBL1918 | Ligand-gated ion channel | 0.115737 |
| Dapagliflozin | Beta-galactosidase | GLB1 | P16278 | CHEMBL2522 | Hydrolase | 0.115737 |
| Dapagliflozin | MAP kinase ERK2 | MAPK1 | P28482 | CHEMBL4040 | Kinase | 0.115737 |
| Dapagliflozin | Endothelin-converting enzyme 1 | ECE1 | P42892 | CHEMBL4791 | Protease | 0.115737 |
| Dapagliflozin | Thrombin | F2 | P00734 | CHEMBL204 | Protease | 0.115737 |
| Dapagliflozin | Thrombin and coagulation factor X | F10 | P00742 | CHEMBL244 | Protease | 0.115737 |
| Dapagliflozin | Cyclin-dependent kinase 1/cyclin B1 | CDK1 CCNB1 | P06493 P14635 | CHEMBL1907602 | Other cytosolic protein | 0.115737 |
| Dapagliflozin | Cyclin-dependent kinase 2/cyclin E1 | CCNE1 CDK2 | P24864 P24941 | CHEMBL1907605 | Kinase | 0.115737 |
| Dapagliflozin | Cyclin-dependent kinase 7/ cyclin H | CDK7 CCNH | P50613 P51946 | CHEMBL2111288 | Other cytosolic protein | 0.115737 |
| Dapagliflozin | CDK9/cyclin T1 | CDK9 CCNT1 | P50750 O60563 | CHEMBL2111389 | Other cytosolic protein | 0.115737 |
| Dapagliflozin | Dual-specificity tyrosine-phosphorylation regulated kinase 1A | DYRK1A | Q13627 | CHEMBL2292 | Kinase | 0.115737 |
| Dapagliflozin | Casein kinase I alpha | CSNK1A1 | P48729 | CHEMBL2793 | Kinase | 0.115737 |
| Dapagliflozin | Cyclin-dependent kinase 2 | CDK2 | P24941 | CHEMBL301 | Kinase | 0.115737 |
| Dapagliflozin | Cyclin-dependent kinase 1 | CDK1 | P06493 | CHEMBL308 | Kinase | 0.115737 |
| Dapagliflozin | MAP kinase ERK1 | MAPK3 | P27361 | CHEMBL3385 | Kinase | 0.115737 |
| Dapagliflozin | Mitogen-activated protein kinase 15 | MAPK15 | Q8TD08 | CHEMBL5198 | Kinase | 0.115737 |
| Dapagliflozin | CaM-kinase kinase beta | CAMKK2 | Q96RR4 | CHEMBL5284 | Kinase | 0.115737 |
| Dapagliflozin | Insulin receptor-related protein | INSRR | P14616 | CHEMBL5483 | Kinase | 0.115737 |
| Dapagliflozin | Maltase-glucoamylase | MGAM | O43451 | CHEMBL2074 | Hydrolase | 0 |
| Dapagliflozin | Sucrase-isomaltase | SI | P14410 | CHEMBL2748 | Enzyme | 0 |
| Dapagliflozin | G-protein coupled receptor 55 | GPR55 | Q9Y2T6 | CHEMBL1075322 | Family A G protein-coupled receptor | 0 |
| Dapagliflozin | G-protein coupled receptor 35 | GPR35 | Q9HC97 | CHEMBL1293267 | Family A G protein-coupled receptor | 0 |
| Dapagliflozin | Type-1 angiotensin II receptor (by homology) | AGTR1 | P30556 | CHEMBL227 | Family A G protein-coupled receptor | 0 |
| Dapagliflozin | Caspase-3 | CASP3 | P42574 | CHEMBL2334 | Protease | 0 |
| Dapagliflozin | Serine/threonine-protein kinase receptor R3 | ACVRL1 | P37023 | CHEMBL5311 | Kinase | 0 |
| Dapagliflozin | Squalene synthetase | FDFT1 | P37268 | CHEMBL3338 | Enzyme | 0 |
| Dapagliflozin | Tyrosine-protein kinase ABL | ABL1 | P00519 | CHEMBL1862 | Kinase | 0 |
| Dapagliflozin | DNA (cytosine-5)-methyltransferase 1 | DNMT1 | P26358 | CHEMBL1993 | Writer | 0 |
| Dapagliflozin | Inhibitor of nuclear factor kappa B kinase beta subunit | IKBKB | O14920 | CHEMBL1991 | Kinase | 0 |
| Dapagliflozin | Caspase-7 | CASP7 | P55210 | CHEMBL3468 | Protease | 0 |
| Dapagliflozin | Galectin-3 | LGALS3 | P17931 | CHEMBL4531 | Other cytosolic protein | 0 |
| Dapagliflozin | Galectin-1 | LGALS1 | P09382 | CHEMBL4915 | Other cytosolic protein | 0 |
| Dapagliflozin | Serine/threonine-protein kinase B-raf | BRAF | P15056 | CHEMBL5145 | Kinase | 0 |
| Dapagliflozin | Serine/threonine-protein kinase ILK-1 | ILK | Q13418 | CHEMBL5247 | Kinase | 0 |
| Dapagliflozin | Nerve growth factor receptor Trk-A | NTRK1 | P04629 | CHEMBL2815 | Kinase | 0 |
| Dapagliflozin | Alcohol dehydrogenase class III | ADH5 | P11766 | CHEMBL4116 | Enzyme | 0 |
| Dapagliflozin | Thymidine kinase, mitochondrial | TK2 | O00142 | CHEMBL4580 | Enzyme | 0 |
| Dapagliflozin | Dipeptidyl peptidase IV | DPP4 | P27487 | CHEMBL284 | Protease | 0 |
| Dapagliflozin | Muscle glycogen synthase | GYS1 | P13807 | CHEMBL4000 | Enzyme | 0 |
| Dapagliflozin | Acyl-CoA desaturase | SCD | O00767 | CHEMBL5555 | Enzyme | 0 |
| Dapagliflozin | c-Jun N-terminal kinase 1 | MAPK8 | P45983 | CHEMBL2276 | Kinase | 0 |
| Dapagliflozin | Tyrosine-protein kinase SRC | SRC | P12931 | CHEMBL267 | Kinase | 0 |
| Dapagliflozin | Ileal bile acid transporter | SLC10A2 | Q12908 | CHEMBL2778 | Electrochemical transporter | 0 |
| Dapagliflozin | Isoleucyl-tRNA synthetase | IARS | P41252 | CHEMBL3235 | Enzyme | 0 |
| Dapagliflozin | Leucyl-tRNA synthetase | LARS | Q9P2J5 | CHEMBL3258 | Enzyme | 0 |
| Dapagliflozin | Protein arginine N-methyltransferase 7 | PRMT7 | Q9NVM4 | CHEMBL3562175 | Writer | 0 |
| Dapagliflozin | Cyclin-dependent kinase 2/cyclin A | CDK2 CCNA1 CCNA2 | P24941 P78396 P20248 | CHEMBL2094128 | Other cytosolic protein | 0 |
|  |  |  |  |  |  |  |
| Canagliflozin | Sodium/glucose cotransporter 2 | SLC5A2 | P31639 | CHEMBL3884 | Electrochemical transporter | 1 |
| Canagliflozin | Sodium/glucose cotransporter 1 | SLC5A1 | P13866 | CHEMBL4979 | Electrochemical transporter | 1 |
| Canagliflozin | Adenosine A1 receptor (by homology) | ADORA1 | P30542 | CHEMBL226 | Family A G protein-coupled receptor | 0.118883 |
| Canagliflozin | Adenosine kinase | ADK | P55263 | CHEMBL3589 | Enzyme | 0.118883 |
| Canagliflozin | Coagulation factor VII/tissue factor | F3 | P13726 | CHEMBL4081 | Surface antigen | 0.118883 |
| Canagliflozin | Adenosine A2a receptor (by homology) | ADORA2A | P29274 | CHEMBL251 | Family A G protein-coupled receptor | 0.118883 |
| Canagliflozin | Adenosine A3 receptor | ADORA3 | P0DMS8 | CHEMBL256 | Family A G protein-coupled receptor | 0.118883 |
| Canagliflozin | Carbonic anhydrase XII | CA12 | O43570 | CHEMBL3242 | Lyase | 0.118883 |
| Canagliflozin | Carbonic anhydrase IX | CA9 | Q16790 | CHEMBL3594 | Lyase | 0.118883 |
| Canagliflozin | Beta-glucosidase | GBA2 | Q9HCG7 | CHEMBL3761 | Enzyme | 0.118883 |
| Canagliflozin | Purinergic receptor P2Y12 (by homology) | P2RY12 | Q9H244 | CHEMBL2001 | Family A G protein-coupled receptor | 0.118883 |
| Canagliflozin | Beta-glucocerebrosidase (by homology) | GBA | P04062 | CHEMBL2179 | Enzyme | 0.118883 |
| Canagliflozin | Equilibrative nucleoside transporter 1 | SLC29A1 | Q99808 | CHEMBL1997 | Electrochemical transporter | 0.118883 |
| Canagliflozin | Sodium/nucleoside cotransporter 2 | SLC28A2 | O43868 | CHEMBL5780 | Electrochemical transporter | 0.118883 |
| Canagliflozin | Glucose transporter (by homology) | SLC2A1 | P11166 | CHEMBL2535 | Electrochemical transporter | 0.118883 |
| Canagliflozin | Glyceraldehyde-3-phosphate dehydrogenase liver | GAPDH | P04406 | CHEMBL2284 | Oxidoreductase | 0.118883 |
| Canagliflozin | Cathepsin L | CTSL | P07711 | CHEMBL3837 | Protease | 0.118883 |
| Canagliflozin | Carbonic anhydrase I | CA1 | P00915 | CHEMBL261 | Lyase | 0.118883 |
| Canagliflozin | Dual specificity mitogen-activated protein kinase kinase 1 | MAP2K1 | Q02750 | CHEMBL3587 | Kinase | 0.118883 |
| Canagliflozin | Serine/threonine-protein kinase Chk1 | CHEK1 | O14757 | CHEMBL4630 | Kinase | 0.118883 |
| Canagliflozin | Carbonic anhydrase II | CA2 | P00918 | CHEMBL205 | Lyase | 0.118883 |
| Canagliflozin | NAD-dependent deacetylase sirtuin 2 | SIRT2 | Q8IXJ6 | CHEMBL4462 | Eraser | 0.118883 |
| Canagliflozin | Uridine phosphorylase 1 (by homology) | UPP1 | Q16831 | CHEMBL4811 | Enzyme | 0.118883 |
| Canagliflozin | Adenosine A2b receptor | ADORA2B | P29275 | CHEMBL255 | Family A G protein-coupled receptor | 0.118883 |
| Canagliflozin | MAP kinase p38 alpha | MAPK14 | Q16539 | CHEMBL260 | Kinase | 0.118883 |
| Canagliflozin | Carbonic anhydrase XIV | CA14 | Q9ULX7 | CHEMBL3510 | Lyase | 0.118883 |
| Canagliflozin | Hepatocyte growth factor receptor | MET | P08581 | CHEMBL3717 | Kinase | 0.118883 |
| Canagliflozin | Muscle glycogen phosphorylase | PYGM | P11217 | CHEMBL3526 | Enzyme | 0.118883 |
| Canagliflozin | Interleukin-1 receptor-associated kinase 4 | IRAK4 | Q9NWZ3 | CHEMBL3778 | Kinase | 0.118883 |
| Canagliflozin | Heat shock cognate 71 kDa protein | HSPA8 | P11142 | CHEMBL1275223 | Other cytosolic protein | 0.118883 |
| Canagliflozin | 78 kDa glucose-regulated protein | HSPA5 | P11021 | CHEMBL1781865 | Unclassified protein | 0.118883 |
| Canagliflozin | Cyclin-dependent kinase 1/cyclin B1 | CDK1 CCNB1 | P06493 P14635 | CHEMBL1907602 | Other cytosolic protein | 0.118883 |
| Canagliflozin | Cyclin-dependent kinase 2/cyclin E1 | CCNE1 CDK2 | P24864 P24941 | CHEMBL1907605 | Kinase | 0.118883 |
| Canagliflozin | Cyclin-dependent kinase 7/ cyclin H | CDK7 CCNH | P50613 P51946 | CHEMBL2111288 | Other cytosolic protein | 0.118883 |
| Canagliflozin | CDK9/cyclin T1 | CDK9 CCNT1 | P50750 O60563 | CHEMBL2111389 | Other cytosolic protein | 0.118883 |
| Canagliflozin | Dual-specificity tyrosine-phosphorylation regulated kinase 1A | DYRK1A | Q13627 | CHEMBL2292 | Kinase | 0.118883 |
| Canagliflozin | Casein kinase I alpha | CSNK1A1 | P48729 | CHEMBL2793 | Kinase | 0.118883 |
| Canagliflozin | Tyrosine-protein kinase JAK2 | JAK2 | O60674 | CHEMBL2971 | Kinase | 0.118883 |
| Canagliflozin | MAP kinase ERK1 | MAPK3 | P27361 | CHEMBL3385 | Kinase | 0.118883 |
| Canagliflozin | MAP kinase ERK2 | MAPK1 | P28482 | CHEMBL4040 | Kinase | 0.118883 |
| Canagliflozin | Mitogen-activated protein kinase 15 | MAPK15 | Q8TD08 | CHEMBL5198 | Kinase | 0.118883 |
| Canagliflozin | CaM-kinase kinase beta | CAMKK2 | Q96RR4 | CHEMBL5284 | Kinase | 0.118883 |
| Canagliflozin | Insulin receptor-related protein | INSRR | P14616 | CHEMBL5483 | Kinase | 0.118883 |
| Canagliflozin | Bcl-2-related protein A1 (by homology) | BCL2A1 | Q16548 | CHEMBL6044 | Unclassified protein | 0.118883 |
| Canagliflozin | Epidermal growth factor receptor erbB1 | EGFR | P00533 | CHEMBL203 | Kinase | 0.118883 |
| Canagliflozin | Serine/threonine-protein kinase B-raf | BRAF | P15056 | CHEMBL5145 | Kinase | 0.118883 |
| Canagliflozin | Bifunctional protein NCOAT | OGA | O60502 | CHEMBL5921 | Enzyme | 0.118883 |
| Canagliflozin | Muscle glycogen synthase | GYS1 | P13807 | CHEMBL4000 | Enzyme | 0.118883 |
| Canagliflozin | Galectin-3 | LGALS3 | P17931 | CHEMBL4531 | Other cytosolic protein | 0.118883 |
| Canagliflozin | Galectin-1 | LGALS1 | P09382 | CHEMBL4915 | Other cytosolic protein | 0.118883 |
| Canagliflozin | Perforin-1 | PRF1 | P14222 | CHEMBL5480 | Other ion channel | 0.118883 |
| Canagliflozin | Phosphodiesterase 5A | PDE5A | O76074 | CHEMBL1827 | Phosphodiesterase | 0.118883 |
| Canagliflozin | Liver glycogen phosphorylase | PYGL | P06737 | CHEMBL2568 | Enzyme | 0.118883 |
| Canagliflozin | Alkaline phosphatase, tissue-nonspecific isozyme | ALPL | P05186 | CHEMBL5979 | Enzyme | 0.118883 |
| Canagliflozin | Induced myeloid leukemia cell differentiation protein Mcl-1 | MCL1 | Q07820 | CHEMBL4361 | Other cytosolic protein | 0.118883 |
| Canagliflozin | Inosine-5'-monophosphate dehydrogenase 2 | IMPDH2 | P12268 | CHEMBL2002 | Oxidoreductase | 0.118883 |
| Canagliflozin | Eukaryotic initiation factor 4A-I | EIF4A1 | P60842 | CHEMBL2052028 | Hydrolase | 0.118883 |
| Canagliflozin | Inosine-5'-monophosphate dehydrogenase 1 | IMPDH1 | P20839 | CHEMBL1822 | Oxidoreductase | 0.118883 |
| Canagliflozin | Squalene synthetase (by homology) | FDFT1 | P37268 | CHEMBL3338 | Enzyme | 0.118883 |
| Canagliflozin | Heat shock protein HSP 90-alpha | HSP90AA1 | P07900 | CHEMBL3880 | Other cytosolic protein | 0.118883 |
| Canagliflozin | Thymidine kinase, mitochondrial | TK2 | O00142 | CHEMBL4580 | Enzyme | 0.118883 |
| Canagliflozin | Beta-secretase 1 | BACE1 | P56817 | CHEMBL4822 | Protease | 0.118883 |
| Canagliflozin | Angiotensin-converting enzyme (by homology) | ACE | P12821 | CHEMBL1808 | Protease | 0.118883 |
| Canagliflozin | Programmed cell death 1 ligand 1 | CD274 | Q9NZQ7 | CHEMBL3580522 | Unclassified protein | 0.118883 |
| Canagliflozin | Protein-tyrosine phosphatase 2C | PTPN11 | Q06124 | CHEMBL3864 | Phosphatase | 0.118883 |
| Canagliflozin | Glutamate [NMDA] receptor PROTEIN | GRIN1 | Q05586 | CHEMBL2015 | Ligand-gated ion channel | 0 |
| Canagliflozin | Matrix metalloproteinase 3 | MMP3 | P08254 | CHEMBL283 | Protease | 0 |
| Canagliflozin | Matrix metalloproteinase 9 | MMP9 | P14780 | CHEMBL321 | Protease | 0 |
| Canagliflozin | Matrix metalloproteinase 1 | MMP1 | P03956 | CHEMBL332 | Protease | 0 |
| Canagliflozin | ADAM17 | ADAM17 | P78536 | CHEMBL3706 | Protease | 0 |
| Canagliflozin | c-Jun N-terminal kinase 1 | MAPK8 | P45983 | CHEMBL2276 | Kinase | 0 |
| Canagliflozin | Dipeptidyl peptidase IV | DPP4 | P27487 | CHEMBL284 | Protease | 0 |
| Canagliflozin | Sodium channel protein type IX alpha subunit | SCN9A | Q15858 | CHEMBL4296 | Voltage-gated ion channel | 0 |
| Canagliflozin | SUMO-activating enzyme subunit 1 | SAE1 | Q9UBE0 | CHEMBL1615388 | Unclassified protein | 0 |
| Canagliflozin | Neprilysin | MME | P08473 | CHEMBL1944 | Protease | 0 |
| Canagliflozin | NEDD8-activating enzyme E1 regulatory subunit | NAE1 | Q13564 | CHEMBL2016431 | Unclassified protein | 0 |
| Canagliflozin | Peptidyl-prolyl cis-trans isomerase NIMA-interacting 1 | PIN1 | Q13526 | CHEMBL2288 | Enzyme | 0 |
| Canagliflozin | Ubiquitin-like modifier-activating enzyme 6 | UBA6 | A0AVT1 | CHEMBL2321622 | Enzyme | 0 |
| Canagliflozin | Ubiquitin-like modifier-activating enzyme 7 | UBA7 | P41226 | CHEMBL2321623 | Enzyme | 0 |
| Canagliflozin | Matrix metalloproteinase 13 | MMP13 | P45452 | CHEMBL280 | Protease | 0 |
| Canagliflozin | Matrix metalloproteinase 2 | MMP2 | P08253 | CHEMBL333 | Protease | 0 |
| Canagliflozin | Matrix metalloproteinase 7 | MMP7 | P09237 | CHEMBL4073 | Protease | 0 |
| Canagliflozin | Endothelin-converting enzyme 1 | ECE1 | P42892 | CHEMBL4791 | Protease | 0 |
| Canagliflozin | Mitogen-activated protein kinase kinase kinase 5 | MAP3K5 | Q99683 | CHEMBL5285 | Kinase | 0 |
| Canagliflozin | Endothelin receptor ET-B | EDNRB | P24530 | CHEMBL1785 | Family A G protein-coupled receptor | 0 |
| Canagliflozin | Integrin alpha-4/beta-1 | ITGB1 ITGA4 | P05556 P13612 | CHEMBL1907599 | Membrane receptor | 0 |
| Canagliflozin | Maltase-glucoamylase | MGAM | O43451 | CHEMBL2074 | Hydrolase | 0 |
| Canagliflozin | Endothelin receptor ET-A (by homology) | EDNRA | P25101 | CHEMBL252 | Family A G protein-coupled receptor | 0 |
| Canagliflozin | Sucrase-isomaltase | SI | P14410 | CHEMBL2748 | Enzyme | 0 |
| Canagliflozin | Voltage-gated N-type calcium channel alpha-1B subunit | CACNA1B | Q00975 | CHEMBL4478 | Voltage-gated ion channel | 0 |
| Canagliflozin | Hexokinase type IV | GCK | P35557 | CHEMBL3820 | Enzyme | 0 |
| Canagliflozin | Fibroblast growth factor receptor 1 | FGFR1 | P11362 | CHEMBL3650 | Kinase | 0 |
| Canagliflozin | Matrix metalloproteinase 12 | MMP12 | P39900 | CHEMBL4393 | Protease | 0 |
| Canagliflozin | Thrombopoietin receptor | MPL | P40238 | CHEMBL1864 | Membrane receptor | 0 |
| Canagliflozin | Platelet-derived growth factor receptor beta | PDGFRB | P09619 | CHEMBL1913 | Kinase | 0 |
| Canagliflozin | Tyrosine-protein kinase LCK | LCK | P06239 | CHEMBL258 | Kinase | 0 |
| Canagliflozin | c-Jun N-terminal kinase 3 | MAPK10 | P53779 | CHEMBL2637 | Kinase | 0 |
| Canagliflozin | Nerve growth factor receptor Trk-A | NTRK1 | P04629 | CHEMBL2815 | Kinase | 0 |
| Canagliflozin | Cyclin-dependent kinase 1 | CDK1 | P06493 | CHEMBL308 | Kinase | 0 |
| Canagliflozin | MAP kinase p38 beta | MAPK11 | Q15759 | CHEMBL3961 | Kinase | 0 |
|  |  |  |  |  |  |  |
| Empagliflozin | Sodium/glucose cotransporter 2 | SLC5A2 | P31639 | CHEMBL3884 | Electrochemical transporter | 1 |
| Empagliflozin | Sodium/glucose cotransporter 1 | SLC5A1 | P13866 | CHEMBL4979 | Electrochemical transporter | 1 |
| Empagliflozin | Sodium/myo-inositol cotransporter 2 | SLC5A11 | Q8WWX8 | CHEMBL1744524 | Electrochemical transporter | 0.440574 |
| Empagliflozin | Low affinity sodium-glucose cotransporter | SLC5A4 | Q9NY91 | CHEMBL1770047 | Electrochemical transporter | 0.440574 |
| Empagliflozin | Equilibrative nucleoside transporter 1 | SLC29A1 | Q99808 | CHEMBL1997 | Electrochemical transporter | 0.118883 |
| Empagliflozin | Adenosine kinase | ADK | P55263 | CHEMBL3589 | Enzyme | 0.118883 |
| Empagliflozin | Serine/threonine-protein kinase PIM1 | PIM1 | P11309 | CHEMBL2147 | Kinase | 0.118883 |
| Empagliflozin | Dual specificity mitogen-activated protein kinase kinase 1 | MAP2K1 | Q02750 | CHEMBL3587 | Kinase | 0.118883 |
| Empagliflozin | Glucose transporter (by homology) | SLC2A1 | P11166 | CHEMBL2535 | Electrochemical transporter | 0.118883 |
| Empagliflozin | Coagulation factor VII/tissue factor | F3 | P13726 | CHEMBL4081 | Surface antigen | 0.118883 |
| Empagliflozin | Phosphodiesterase 5A | PDE5A | O76074 | CHEMBL1827 | Phosphodiesterase | 0.118883 |
| Empagliflozin | Neprilysin (by homology) | MME | P08473 | CHEMBL1944 | Protease | 0.118883 |
| Empagliflozin | Adenosine A2a receptor (by homology) | ADORA2A | P29274 | CHEMBL251 | Family A G protein-coupled receptor | 0.118883 |
| Empagliflozin | Beta-glucocerebrosidase | GBA | P04062 | CHEMBL2179 | Enzyme | 0.118883 |
| Empagliflozin | NAD-dependent deacetylase sirtuin 2 | SIRT2 | Q8IXJ6 | CHEMBL4462 | Eraser | 0.118883 |
| Empagliflozin | Purinergic receptor P2Y12 | P2RY12 | Q9H244 | CHEMBL2001 | Family A G protein-coupled receptor | 0.118883 |
| Empagliflozin | Uridine phosphorylase 1 (by homology) | UPP1 | Q16831 | CHEMBL4811 | Enzyme | 0 |
| Empagliflozin | Hexokinase type II | HK2 | P52789 | CHEMBL2640 | Enzyme | 0 |
| Empagliflozin | Hexokinase type I | HK1 | P19367 | CHEMBL2688 | Enzyme | 0 |
| Empagliflozin | Beta-glucosidase | GBA2 | Q9HCG7 | CHEMBL3761 | Enzyme | 0 |
| Empagliflozin | Ceramide glucosyltransferase | UGCG | Q16739 | CHEMBL2063 | Transferase | 0 |
| Empagliflozin | Maltase-glucoamylase | MGAM | O43451 | CHEMBL2074 | Hydrolase | 0 |
| Empagliflozin | Sucrase-isomaltase | SI | P14410 | CHEMBL2748 | Enzyme | 0 |
| Empagliflozin | Platelet-derived growth factor receptor beta | PDGFRB | P09619 | CHEMBL1913 | Kinase | 0 |
| Empagliflozin | Epidermal growth factor receptor erbB1 | EGFR | P00533 | CHEMBL203 | Kinase | 0 |
| Empagliflozin | Tyrosine-protein kinase LCK | LCK | P06239 | CHEMBL258 | Kinase | 0 |
| Empagliflozin | c-Jun N-terminal kinase 3 | MAPK10 | P53779 | CHEMBL2637 | Kinase | 0 |
| Empagliflozin | MAP kinase p38 beta | MAPK11 | Q15759 | CHEMBL3961 | Kinase | 0 |
| Empagliflozin | Ephrin type-A receptor 5 | EPHA5 | P54756 | CHEMBL3987 | Kinase | 0 |
| Empagliflozin | c-Jun N-terminal kinase 2 | MAPK9 | P45984 | CHEMBL4179 | Kinase | 0 |
| Empagliflozin | Serine/threonine-protein kinase GAK | GAK | O14976 | CHEMBL4355 | Kinase | 0 |
| Empagliflozin | Sodium/nucleoside cotransporter 2 | SLC28A2 | O43868 | CHEMBL5780 | Electrochemical transporter | 0 |
| Empagliflozin | Dihydrofolate reductase | DHFR | P00374 | CHEMBL202 | Oxidoreductase | 0 |
| Empagliflozin | Liver glycogen phosphorylase | PYGL | P06737 | CHEMBL2568 | Enzyme | 0 |
| Empagliflozin | Adenosine A2b receptor | ADORA2B | P29275 | CHEMBL255 | Family A G protein-coupled receptor | 0 |
| Empagliflozin | Insulin-like growth factor binding protein 3 | IGFBP3 | P17936 | CHEMBL3997 | Secreted protein | 0 |
| Empagliflozin | DNA topoisomerase I | TOP1 | P11387 | CHEMBL1781 | Isomerase | 0 |
| Empagliflozin | Interleukin-1 receptor-associated kinase 4 | IRAK4 | Q9NWZ3 | CHEMBL3778 | Kinase | 0 |
| Empagliflozin | Endothelin-converting enzyme 1 | ECE1 | P42892 | CHEMBL4791 | Protease | 0 |
| Empagliflozin | Perforin-1 | PRF1 | P14222 | CHEMBL5480 | Other ion channel | 0 |
| Empagliflozin | GABA receptor alpha-5 subunit | GABRA5 | P31644 | CHEMBL5112 | Ligand-gated ion channel | 0 |
| Empagliflozin | Serine/threonine-protein kinase PLK1 | PLK1 | P53350 | CHEMBL3024 | Kinase | 0 |
| Empagliflozin | Serine/threonine-protein kinase PLK3 | PLK3 | Q9H4B4 | CHEMBL4897 | Kinase | 0 |
| Empagliflozin | Type-1 angiotensin II receptor (by homology) | AGTR1 | P30556 | CHEMBL227 | Family A G protein-coupled receptor | 0 |
| Empagliflozin | Muscle glycogen synthase | GYS1 | P13807 | CHEMBL4000 | Enzyme | 0 |
| Empagliflozin | Chymase | CMA1 | P23946 | CHEMBL4068 | Protease | 0 |
| Empagliflozin | MAP kinase signal-integrating kinase 2 | MKNK2 | Q9HBH9 | CHEMBL4204 | Kinase | 0 |
| Empagliflozin | Serine/threonine-protein kinase ILK-1 | ILK | Q13418 | CHEMBL5247 | Kinase | 0 |
| Empagliflozin | Splicing factor 3B subunit 3 | SF3B3 | Q15393 | CHEMBL1250378 | Unclassified protein | 0 |
| Empagliflozin | Matrix metalloproteinase 13 | MMP13 | P45452 | CHEMBL280 | Protease | 0 |
| Empagliflozin | Matrix metalloproteinase 8 | MMP8 | P22894 | CHEMBL4588 | Protease | 0 |
| Empagliflozin | Tyrosine-protein kinase SYK | SYK | P43405 | CHEMBL2599 | Kinase | 0 |
| Empagliflozin | Tyrosine-protein kinase JAK2 | JAK2 | O60674 | CHEMBL2971 | Kinase | 0 |
| Empagliflozin | Glutamate receptor ionotropic kainate 1 | GRIK1 | P39086 | CHEMBL1918 | Ligand-gated ion channel | 0 |
| Empagliflozin | MAP kinase ERK2 | MAPK1 | P28482 | CHEMBL4040 | Kinase | 0 |
| Empagliflozin | Dopamine D4 receptor | DRD4 | P21917 | CHEMBL219 | Family A G protein-coupled receptor | 0 |
| Empagliflozin | Mitogen-activated protein kinase kinase kinase 14 | MAP3K14 | Q99558 | CHEMBL5888 | Kinase | 0 |
| Empagliflozin | Glyceraldehyde-3-phosphate dehydrogenase liver | GAPDH | P04406 | CHEMBL2284 | Oxidoreductase | 0 |
| Empagliflozin | Serine/threonine-protein kinase Chk1 | CHEK1 | O14757 | CHEMBL4630 | Kinase | 0 |
| Empagliflozin | Lysine-specific demethylase 3A | KDM3A | Q9Y4C1 | CHEMBL1938209 | Eraser | 0 |
| Empagliflozin | Thrombin | F2 | P00734 | CHEMBL204 | Protease | 0 |
| Empagliflozin | Endoplasmic reticulum mannosyl-oligosaccharide 1,2-alpha-mannosidase | MAN1B1 | Q9UKM7 | CHEMBL2308 | Enzyme | 0 |
| Empagliflozin | Lysosomal alpha-glucosidase (by homology) | GAA | P10253 | CHEMBL2608 | Hydrolase | 0 |
| Empagliflozin | Ileal bile acid transporter | SLC10A2 | Q12908 | CHEMBL2778 | Electrochemical transporter | 0 |
| Empagliflozin | Lysine-specific demethylase 5B | KDM5B | Q9UGL1 | CHEMBL3774295 | Eraser | 0 |
| Empagliflozin | Alpha-mannosidase 2A1 | MAN2A1 | Q16706 | CHEMBL4056 | Enzyme | 0 |
| Empagliflozin | Lysine-specific demethylase 4D | KDM4D | Q6B0I6 | CHEMBL6138 | Eraser | 0 |
| Empagliflozin | Lysine-specific demethylase 4C | KDM4C | Q9H3R0 | CHEMBL6175 | Eraser | 0 |
| Empagliflozin | Beta-galactosidase | GLB1 | P16278 | CHEMBL2522 | Hydrolase | 0 |
| Empagliflozin | Cyclin-dependent kinase 2 | CDK2 | P24941 | CHEMBL301 | Kinase | 0 |
| Empagliflozin | Cyclin-dependent kinase 1 | CDK1 | P06493 | CHEMBL308 | Kinase | 0 |
| Empagliflozin | Muscle glycogen phosphorylase | PYGM | P11217 | CHEMBL3526 | Enzyme | 0 |
| Empagliflozin | Hydroxycarboxylic acid receptor 2 | HCAR2 | Q8TDS4 | CHEMBL3785 | Family A G protein-coupled receptor | 0 |
| Empagliflozin | Galectin-3 | LGALS3 | P17931 | CHEMBL4531 | Other cytosolic protein | 0 |
| Empagliflozin | Galectin-1 | LGALS1 | P09382 | CHEMBL4915 | Other cytosolic protein | 0 |
| Empagliflozin | Receptor protein-tyrosine kinase erbB-2 | ERBB2 | P04626 | CHEMBL1824 | Kinase | 0 |
| Empagliflozin | DNA polymerase alpha subunit | POLA1 | P09884 | CHEMBL1828 | Transferase | 0 |
| Empagliflozin | Matrix metalloproteinase 3 | MMP3 | P08254 | CHEMBL283 | Protease | 0 |
| Empagliflozin | Matrix metalloproteinase 1 | MMP1 | P03956 | CHEMBL332 | Protease | 0 |
| Empagliflozin | ADAM17 | ADAM17 | P78536 | CHEMBL3706 | Protease | 0 |
| Empagliflozin | Cathepsin L | CTSL | P07711 | CHEMBL3837 | Protease | 0 |
| Empagliflozin | Integrin alpha-4/beta-1 | ITGB1 ITGA4 | P05556 P13612 | CHEMBL1907599 | Membrane receptor | 0 |
| Empagliflozin | Dipeptidyl peptidase IV | DPP4 | P27487 | CHEMBL284 | Protease | 0 |
| Empagliflozin | ALK tyrosine kinase receptor | ALK | Q9UM73 | CHEMBL4247 | Kinase | 0 |
| Empagliflozin | Angiotensin-converting enzyme | ACE | P12821 | CHEMBL1808 | Protease | 0 |
| Empagliflozin | Interleukin-8 receptor B | CXCR2 | P25025 | CHEMBL2434 | Family A G protein-coupled receptor | 0 |
| Empagliflozin | Carbonic anhydrase XIV | CA14 | Q9ULX7 | CHEMBL3510 | Lyase | 0 |
| Empagliflozin | Interferon-induced, double-stranded RNA-activated protein kinase | EIF2AK2 | P19525 | CHEMBL5785 | Kinase | 0 |
| Empagliflozin | 6-phosphofructo-2-kinase/fructose-2,6-bisphosphatase 3 | PFKFB3 | Q16875 | CHEMBL2331053 | Enzyme | 0 |
| Empagliflozin | Heat shock protein HSP 90-alpha | HSP90AA1 | P07900 | CHEMBL3880 | Other cytosolic protein | 0 |
| Empagliflozin | Thymidine kinase, mitochondrial | TK2 | O00142 | CHEMBL4580 | Enzyme | 0 |
| Empagliflozin | TNF-alpha | TNF | P01375 | CHEMBL1825 | Secreted protein | 0 |
| Empagliflozin | Thromboxane A2 receptor | TBXA2R | P21731 | CHEMBL2069 | Family A G protein-coupled receptor | 0 |
| Empagliflozin | G-protein coupled receptor kinase 2 | GRK2 | P25098 | CHEMBL4079 | Kinase | 0 |
| Empagliflozin | Alcohol dehydrogenase class III | ADH5 | P11766 | CHEMBL4116 | Enzyme | 0 |
| Empagliflozin | Runt-related transcription factor 1/Core-binding factor subunit beta | CBFB | Q13951 | CHEMBL1615386 | Unclassified protein | 0 |
| Empagliflozin | Macrophage colony stimulating factor receptor (by homology) | CSF1R | P07333 | CHEMBL1844 | Kinase | 0 |
| Empagliflozin | Alpha-L-fucosidase I | FUCA1 | P04066 | CHEMBL4176 | Enzyme | 0 |
| Empagliflozin | Focal adhesion kinase 1 | PTK2 | Q05397 | CHEMBL2695 | Kinase | 0 |
| Empagliflozin | Fibroblast growth factor receptor 3 | FGFR3 | P22607 | CHEMBL2742 | Kinase | 0 |
|  |  |  |  |  |  |  |
| Ertugliflozin | Low affinity sodium-glucose cotransporter | SLC5A4 | Q9NY91 | CHEMBL1770047 | Electrochemical transporter | 1 |
| Ertugliflozin | Sodium/glucose cotransporter 1 | SLC5A1 | P13866 | CHEMBL4979 | Electrochemical transporter | 1 |
| Ertugliflozin | Sodium/glucose cotransporter 2 (by homology) | SLC5A2 | P31639 | CHEMBL3884 | Electrochemical transporter | 0.959265 |
| Ertugliflozin | Sodium/myo-inositol cotransporter 2 | SLC5A11 | Q8WWX8 | CHEMBL1744524 | Electrochemical transporter | 0.23112 |
| Ertugliflozin | Adenosine A2a receptor | ADORA2A | P29274 | CHEMBL251 | Family A G protein-coupled receptor | 0.106166 |
| Ertugliflozin | Adenosine A3 receptor | ADORA3 | P0DMS8 | CHEMBL256 | Family A G protein-coupled receptor | 0.106166 |
| Ertugliflozin | Adenosine kinase | ADK | P55263 | CHEMBL3589 | Enzyme | 0.106166 |
| Ertugliflozin | Equilibrative nucleoside transporter 1 | SLC29A1 | Q99808 | CHEMBL1997 | Electrochemical transporter | 0.106166 |
| Ertugliflozin | Carbonic anhydrase XII | CA12 | O43570 | CHEMBL3242 | Lyase | 0.106166 |
| Ertugliflozin | Carbonic anhydrase IX | CA9 | Q16790 | CHEMBL3594 | Lyase | 0.106166 |
| Ertugliflozin | Carbonic anhydrase II | CA2 | P00918 | CHEMBL205 | Lyase | 0.106166 |
| Ertugliflozin | Carbonic anhydrase I | CA1 | P00915 | CHEMBL261 | Lyase | 0.106166 |
| Ertugliflozin | Glucose transporter (by homology) | SLC2A1 | P11166 | CHEMBL2535 | Electrochemical transporter | 0.106166 |
| Ertugliflozin | Epidermal growth factor receptor erbB1 | EGFR | P00533 | CHEMBL203 | Kinase | 0.106166 |
| Ertugliflozin | Beta-glucocerebrosidase | GBA | P04062 | CHEMBL2179 | Enzyme | 0.106166 |
| Ertugliflozin | Dual specificity mitogen-activated protein kinase kinase 1 | MAP2K1 | Q02750 | CHEMBL3587 | Kinase | 0.106166 |
| Ertugliflozin | Phosphodiesterase 5A | PDE5A | O76074 | CHEMBL1827 | Phosphodiesterase | 0.106166 |
| Ertugliflozin | Matrix metalloproteinase 3 | MMP3 | P08254 | CHEMBL283 | Protease | 0.106166 |
| Ertugliflozin | Matrix metalloproteinase 9 | MMP9 | P14780 | CHEMBL321 | Protease | 0.106166 |
| Ertugliflozin | Matrix metalloproteinase 1 | MMP1 | P03956 | CHEMBL332 | Protease | 0.106166 |
| Ertugliflozin | Muscle glycogen phosphorylase | PYGM | P11217 | CHEMBL3526 | Enzyme | 0.106166 |
| Ertugliflozin | ADAM17 | ADAM17 | P78536 | CHEMBL3706 | Protease | 0.106166 |
| Ertugliflozin | Heat shock cognate 71 kDa protein | HSPA8 | P11142 | CHEMBL1275223 | Other cytosolic protein | 0.106166 |
| Ertugliflozin | Adenosine A2b receptor | ADORA2B | P29275 | CHEMBL255 | Family A G protein-coupled receptor | 0.106166 |
| Ertugliflozin | Hexokinase type II | HK2 | P52789 | CHEMBL2640 | Enzyme | 0.106166 |
| Ertugliflozin | Hexokinase type I | HK1 | P19367 | CHEMBL2688 | Enzyme | 0.106166 |
| Ertugliflozin | 78 kDa glucose-regulated protein | HSPA5 | P11021 | CHEMBL1781865 | Unclassified protein | 0.106166 |
| Ertugliflozin | Coagulation factor VII/tissue factor | F3 | P13726 | CHEMBL4081 | Surface antigen | 0.106166 |
| Ertugliflozin | Sodium/nucleoside cotransporter 2 | SLC28A2 | O43868 | CHEMBL5780 | Electrochemical transporter | 0.106166 |
| Ertugliflozin | Adenosine A1 receptor | ADORA1 | P30542 | CHEMBL226 | Family A G protein-coupled receptor | 0.106166 |
| Ertugliflozin | MAP kinase p38 alpha | MAPK14 | Q16539 | CHEMBL260 | Kinase | 0.106166 |
| Ertugliflozin | Uridine phosphorylase 1 (by homology) | UPP1 | Q16831 | CHEMBL4811 | Enzyme | 0.106166 |
| Ertugliflozin | Carbonic anhydrase XIV | CA14 | Q9ULX7 | CHEMBL3510 | Lyase | 0.106166 |
| Ertugliflozin | Purinergic receptor P2Y12 (by homology) | P2RY12 | Q9H244 | CHEMBL2001 | Family A G protein-coupled receptor | 0.106166 |
| Ertugliflozin | GABA receptor alpha-5 subunit | GABRA5 | P31644 | CHEMBL5112 | Ligand-gated ion channel | 0.106166 |
| Ertugliflozin | Type-1 angiotensin II receptor (by homology) | AGTR1 | P30556 | CHEMBL227 | Family A G protein-coupled receptor | 0.106166 |
| Ertugliflozin | Glyceraldehyde-3-phosphate dehydrogenase liver | GAPDH | P04406 | CHEMBL2284 | Oxidoreductase | 0.106166 |
| Ertugliflozin | Bifunctional protein NCOAT | OGA | O60502 | CHEMBL5921 | Enzyme | 0.106166 |
| Ertugliflozin | 6-O-methylguanine-DNA methyltransferase | MGMT | P16455 | CHEMBL2864 | Enzyme | 0.106166 |
| Ertugliflozin | Beta-mannosidase | MANBA | O00462 | CHEMBL3903 | Enzyme | 0.106166 |
| Ertugliflozin | Glycogen synthase kinase-3 beta | GSK3B | P49841 | CHEMBL262 | Kinase | 0.106166 |
| Ertugliflozin | Tyrosine-protein kinase SRC | SRC | P12931 | CHEMBL267 | Kinase | 0.106166 |
| Ertugliflozin | Matrix metalloproteinase 13 | MMP13 | P45452 | CHEMBL280 | Protease | 0.106166 |
| Ertugliflozin | MAP kinase ERK2 | MAPK1 | P28482 | CHEMBL4040 | Kinase | 0.106166 |
| Ertugliflozin | Matrix metalloproteinase 7 | MMP7 | P09237 | CHEMBL4073 | Protease | 0.106166 |
| Ertugliflozin | Matrix metalloproteinase 12 | MMP12 | P39900 | CHEMBL4393 | Protease | 0.106166 |
| Ertugliflozin | Matrix metalloproteinase 8 | MMP8 | P22894 | CHEMBL4588 | Protease | 0.106166 |
| Ertugliflozin | Neprilysin | MME | P08473 | CHEMBL1944 | Protease | 0.106166 |
| Ertugliflozin | Liver glycogen phosphorylase | PYGL | P06737 | CHEMBL2568 | Enzyme | 0.106166 |
| Ertugliflozin | Alpha-L-fucosidase I | FUCA1 | P04066 | CHEMBL4176 | Enzyme | 0.106166 |
| Ertugliflozin | Endothelin-converting enzyme 1 | ECE1 | P42892 | CHEMBL4791 | Protease | 0.106166 |
| Ertugliflozin | Fibroblast growth factor receptor 1 | FGFR1 | P11362 | CHEMBL3650 | Kinase | 0.106166 |
| Ertugliflozin | Platelet-derived growth factor receptor beta | PDGFRB | P09619 | CHEMBL1913 | Kinase | 0.106166 |
| Ertugliflozin | Glutamate receptor ionotropic kainate 1 | GRIK1 | P39086 | CHEMBL1918 | Ligand-gated ion channel | 0.106166 |
| Ertugliflozin | GTPase NRas | NRAS | P01111 | CHEMBL2079845 | Unclassified protein | 0.106166 |
| Ertugliflozin | Beta-1 adrenergic receptor (by homology) | ADRB1 | P08588 | CHEMBL213 | Family A G protein-coupled receptor | 0.106166 |
| Ertugliflozin | Tyrosine-protein kinase LCK | LCK | P06239 | CHEMBL258 | Kinase | 0.106166 |
| Ertugliflozin | c-Jun N-terminal kinase 3 | MAPK10 | P53779 | CHEMBL2637 | Kinase | 0.106166 |
| Ertugliflozin | MAP kinase p38 beta | MAPK11 | Q15759 | CHEMBL3961 | Kinase | 0.106166 |
| Ertugliflozin | Ephrin type-A receptor 5 | EPHA5 | P54756 | CHEMBL3987 | Kinase | 0.106166 |
| Ertugliflozin | Serine/threonine-protein kinase GAK | GAK | O14976 | CHEMBL4355 | Kinase | 0.106166 |
| Ertugliflozin | DNA topoisomerase I | TOP1 | P11387 | CHEMBL1781 | Isomerase | 0 |
| Ertugliflozin | DNA (cytosine-5)-methyltransferase 1 | DNMT1 | P26358 | CHEMBL1993 | Writer | 0 |
| Ertugliflozin | Cathepsin L | CTSL | P07711 | CHEMBL3837 | Protease | 0 |
| Ertugliflozin | Tyrosine-protein kinase ABL | ABL1 | P00519 | CHEMBL1862 | Kinase | 0 |
| Ertugliflozin | Stem cell growth factor receptor | KIT | P10721 | CHEMBL1936 | Kinase | 0 |
| Ertugliflozin | Platelet-derived growth factor receptor | PDGFRA PDGFRB | P16234 P09619 | CHEMBL2095189 | Kinase | 0 |
| Ertugliflozin | Interleukin-1 receptor-associated kinase 4 | IRAK4 | Q9NWZ3 | CHEMBL3778 | Kinase | 0 |
| Ertugliflozin | Cyclin-dependent kinase 2 | CDK2 | P24941 | CHEMBL301 | Kinase | 0 |
| Ertugliflozin | Cyclin-dependent kinase 1 | CDK1 | P06493 | CHEMBL308 | Kinase | 0 |
| Ertugliflozin | Alcohol dehydrogenase class III | ADH5 | P11766 | CHEMBL4116 | Enzyme | 0 |
| Ertugliflozin | Tyrosine-protein kinase JAK2 | JAK2 | O60674 | CHEMBL2971 | Kinase | 0 |
| Ertugliflozin | Maltase-glucoamylase | MGAM | O43451 | CHEMBL2074 | Hydrolase | 0 |
| Ertugliflozin | Sucrase-isomaltase | SI | P14410 | CHEMBL2748 | Enzyme | 0 |
| Ertugliflozin | Beta-glucosidase | GBA2 | Q9HCG7 | CHEMBL3761 | Enzyme | 0 |
| Ertugliflozin | Histone acetyltransferase p300 | EP300 | Q09472 | CHEMBL3784 | Writer | 0 |
| Ertugliflozin | Thymidine kinase, mitochondrial | TK2 | O00142 | CHEMBL4580 | Enzyme | 0 |
| Ertugliflozin | Tyrosine-protein kinase ITK/TSK | ITK | Q08881 | CHEMBL2959 | Kinase | 0 |
| Ertugliflozin | Endoplasmic reticulum mannosyl-oligosaccharide 1,2-alpha-mannosidase | MAN1B1 | Q9UKM7 | CHEMBL2308 | Enzyme | 0 |
| Ertugliflozin | Alpha-mannosidase 2A1 | MAN2A1 | Q16706 | CHEMBL4056 | Enzyme | 0 |
| Ertugliflozin | Glutamate [NMDA] receptor PROTEIN | GRIN1 | Q05586 | CHEMBL2015 | Ligand-gated ion channel | 0 |
| Ertugliflozin | Serine/threonine-protein kinase Chk1 | CHEK1 | O14757 | CHEMBL4630 | Kinase | 0 |
| Ertugliflozin | Lysosomal protective protein | CTSA | P10619 | CHEMBL6115 | Protease | 0 |
| Ertugliflozin | Hexokinase type IV | GCK | P35557 | CHEMBL3820 | Enzyme | 0 |
| Ertugliflozin | Acyl-CoA desaturase | SCD | O00767 | CHEMBL5555 | Enzyme | 0 |
| Ertugliflozin | Chymase | CMA1 | P23946 | CHEMBL4068 | Protease | 0 |
| Ertugliflozin | Thymidine phosphorylase | TYMP | P19971 | CHEMBL3106 | Enzyme | 0 |
| Ertugliflozin | Adenosine deaminase | ADA | P00813 | CHEMBL1910 | Hydrolase | 0 |
| Ertugliflozin | Caspase-3 | CASP3 | P42574 | CHEMBL2334 | Protease | 0 |
| Ertugliflozin | Valyl-tRNA synthetase 2 | VARS | P26640 | CHEMBL2612 | Enzyme | 0 |
| Ertugliflozin | Leucyl-tRNA synthetase | LARS | Q9P2J5 | CHEMBL3258 | Enzyme | 0 |
| Ertugliflozin | Cholecystokinin A receptor | CCKAR | P32238 | CHEMBL1901 | Family A G protein-coupled receptor | 0 |
| Ertugliflozin | Cholecystokinin B receptor | CCKBR | P32239 | CHEMBL298 | Family A G protein-coupled receptor | 0 |
| Ertugliflozin | Hydroxycarboxylic acid receptor 2 | HCAR2 | Q8TDS4 | CHEMBL3785 | Family A G protein-coupled receptor | 0 |
| Ertugliflozin | Apoptosis regulator Bcl-X | BCL2L1 | Q07817 | CHEMBL4625 | Other ion channel | 0 |
| Ertugliflozin | Inosine-5'-monophosphate dehydrogenase 1 | IMPDH1 | P20839 | CHEMBL1822 | Oxidoreductase | 0 |
| Ertugliflozin | Inosine-5'-monophosphate dehydrogenase 2 | IMPDH2 | P12268 | CHEMBL2002 | Oxidoreductase | 0 |
| Ertugliflozin | c-Jun N-terminal kinase 1 | MAPK8 | P45983 | CHEMBL2276 | Kinase | 0 |
| Ertugliflozin | Ribosomal protein S6 kinase alpha 3 | RPS6KA3 | P51812 | CHEMBL2345 | Kinase | 0 |
| Ertugliflozin | Ribosomal protein S6 kinase alpha 4 | RPS6KA4 | O75676 | CHEMBL3125 | Kinase | 0 |
