# Supplementary material for: Exploring the Pleiotropic Genes and Therapeutic Targets Associated with Heart Failure and Chronic Kidney Disease by Integrating metaCCA and SGLT2 Inhibitors' Target Prediction
Source: Biomed Res Int. 2021 Sep 8;2021:4229194. doi: 10.1155/2021/4229194 (PMC8443964; doi:10.1155/2021/4229194)
Supplement: Supplementary 2 — Table S2: DrugBank database—four SGLT2 inhibitors. [file 4229194.f2.docx]

| SGLT2 inhibitors | Type | Name | Gene name | Uniprot ID |
| --- | --- | --- | --- | --- |
| Dapagliflozin | target | Sodium/glucose cotransporter 2 | SLC5A2 | P31639 |
| Dapagliflozin | transporter | P-glycoprotein 1 | ABCB1 | P08183 |
| Dapagliflozin | enzyme | Cytochrome P450 1A1 | CYP1A1 | P04798 |
| Dapagliflozin | enzyme | Cytochrome P450 1A2 | CYP1A2 | P05177 |
| Dapagliflozin | enzyme | Cytochrome P450 2C9 | CYP2C9 | P11712 |
| Dapagliflozin | enzyme | UDP-glucuronosyltransferase 2B7 | UGT2B7 | P16662 |
| Dapagliflozin | enzyme | Cytochrome P450 2A6 | CYP2A6 | P11509 |
| Dapagliflozin | enzyme | Cytochrome P450 2D6 | CYP2D6 | P10635 |
| Dapagliflozin | enzyme | Cytochrome P450 3A4 | CYP3A4 | P08684 |
| Dapagliflozin | enzyme | UDP-glucuronosyltransferase 1-9 | UGT1A9 | O60656 |
| Dapagliflozin | enzyme | UDP-glucuronosyltransferase 2B4 | UGT2B4 | P06133 |
|  |  |  |  |  |
| Canagliflozin | carrier | Alpha-1-acid glycoprotein 1 | ORM1 | P02763 |
| Canagliflozin | enzyme | Cytochrome P450 3A4 | CYP3A4 | P08684 |
| Canagliflozin | transporter | P-glycoprotein 1 | ABCB2 | P08184 |
| Canagliflozin | enzyme | UDP-glucuronosyltransferase 1-9 | UGT1A9 | O60656 |
| Canagliflozin | enzyme | UDP-glucuronosyltransferase 2B4 | UGT2B5 | P06133 |
| Canagliflozin | target | Sodium/glucose cotransporter 2 | SLC5A2 | P31639 |
| Canagliflozin | transporter | Canalicular multispecific organic anion transporter 1 | ABCC2 | Q92887 |
| Canagliflozin | transporter | ATP-binding cassette sub-family G member 2 | ABCG2 | Q9UNQ0 |
|  |  |  |  |  |
| Empagliflozin | enzyme | UDP-glucuronosyltransferase 2B7 | UGT2B7 | P16662 |
| Empagliflozin | transporter | ATP-binding cassette sub-family G member 2 | ABCG2 | Q9UNQ0 |
| Empagliflozin | transporter | Solute carrier organic anion transporter family member 1B3 | SLCO1B3 | Q9NPD5 |
| Empagliflozin | target | Sodium/glucose cotransporter 2 | SLC5A2 | P31639 |
| Empagliflozin | enzyme | UDP-glucuronosyltransferase 1-3 | UGT1A3 | P35503 |
| Empagliflozin | enzyme | UDP-glucuronosyltransferase 1-8 | UGT1A8 | Q9HAW9 |
| Empagliflozin | enzyme | UDP-glucuronosyltransferase 1-9 | UGT1A9 | O60656 |
| Empagliflozin | transporter | P-glycoprotein 1 | ABCB1 | P08183 |
| Empagliflozin | transporter | Solute carrier family 22 member 8 | SLC22A8 | Q8TCC7 |
| Empagliflozin | transporter | Solute carrier organic anion transporter family member 1B1 | SLCO1B1 | Q9Y6L6 |
|  |  |  |  |  |
| Ertugliflozin | transporter | ATP-binding cassette sub-family G member 2 | ABCG2 | Q9UNQ0 |
| Ertugliflozin | enzyme | UDP-glucuronosyltransferase 2B7 | UGT2B7 | P16662 |
| Ertugliflozin | transporter | P-glycoprotein 1 | ABCB1 | P08183 |
| Ertugliflozin | enzyme | UDP-glucuronosyltransferase 1-9 | UGT1A9 | O60656 |
| Ertugliflozin | target | Sodium/glucose cotransporter 2 | SLC5A2 | P31639 |
| Ertugliflozin | carrier | Serum albumin | ALB | P02768 |
| Ertugliflozin | enzyme | UDP-glucuronosyltransferase 1-1 | UGT1A1 | P22309 |
| Ertugliflozin | enzyme | UDP-glucuronosyltransferase 1-4 | UGT1A4 | P22310 |
