# Supplementary material for: Exploring the Pleiotropic Genes and Therapeutic Targets Associated with Heart Failure and Chronic Kidney Disease by Integrating metaCCA and SGLT2 Inhibitors' Target Prediction
Source: Biomed Res Int. 2021 Sep 8;2021:4229194. doi: 10.1155/2021/4229194 (PMC8443964; doi:10.1155/2021/4229194)
Supplement: Supplementary 3 — Table S3: 169 putative pleiotropic genes. [file 4229194.f3.docx]

| **Gene** | **MetaCCA P value** | **HF VEGAS2 P-value** | **CKD VEGAS2 P-value** | **HF-TWAS P-value** | **CKD-TWAS P-value** | **genetype** |
| --- | --- | --- | --- | --- | --- | --- |
| USP10 | 0.00E+00 | 2.07E-01 | 1.27E-04 | 7.32E-01 | 9.89E-01 | comfirm |
| FOXO1 | 0.00E+00 | 3.74E-01 | 4.69E-03 | #N/A | #N/A | comfirm |
| GDA | 1.81E-302 | 9.80E-01 | 3.30E-03 | #N/A | #N/A | comfirm |
| PDE3B | 4.35E-291 | 5.81E-01 | 8.47E-03 | #N/A | #N/A | comfirm |
| USP2 | 8.20E-286 | 3.59E-03 | 2.77E-01 | 4.44E-01 | 8.98E-01 | comfirm |
| TRPC3 | 4.36E-205 | 7.99E-04 | 5.11E-01 | #N/A | #N/A | comfirm |
| FTO | 7.55E-147 | 8.80E-05 | 9.80E-04 | 9.12E-01 | 2.68E-01 | comfirm |
| PGR | 3.64E-141 | 8.01E-03 | 5.82E-01 | #N/A | #N/A | comfirm |
| AKT2 | 1.42E-111 | 4.45E-01 | 8.90E-03 | 9.87E-01 | 9.37E-01 | comfirm |
| XPC | 1.96E-108 | 7.87E-01 | 3.04E-04 | 9.01E-01 | 9.02E-01 | comfirm |
| PDGFC | 2.54E-89 | 2.47E-01 | 6.42E-03 | 9.73E-01 | 3.67E-01 | comfirm |
| LMF1 | 7.98E-83 | 4.09E-03 | 6.43E-01 | 7.34E-01 | 8.58E-01 | comfirm |
| DTNB | 1.89E-49 | 5.05E-01 | 8.50E-03 | 9.81E-01 | 9.38E-01 | comfirm |
| PPARGC1A | 4.67E-41 | 6.07E-01 | 6.19E-03 | #N/A | #N/A | comfirm |
| SLC39A8 | 1.29E-27 | 5.78E-03 | 4.43E-03 | 6.33E-01 | 7.88E-01 | comfirm |
| TSHR | 6.79E-22 | 5.76E-03 | 3.18E-01 | 9.61E-01 | 8.67E-01 | comfirm |
| CUX2 | 6.15E-21 | 1.87E-02 | 9.70E-03 | #N/A | #N/A | comfirm |
| PRKAG2 | 8.00E-21 | 8.42E-01 | 1.00E-06 | #N/A | #N/A | comfirm |
| KCNQ1 | 3.01E-19 | 5.92E-01 | 7.48E-03 | 9.60E-01 | 8.96E-01 | comfirm |
| PAX8 | 5.59E-19 | 6.58E-01 | 3.80E-05 | 8.98E-01 | 2.48E-02 | comfirm |
| UMOD | 7.82E-19 | 6.56E-01 | 1.00E-06 | #N/A | #N/A | comfirm |
| MICA | 6.83E-18 | 2.39E-04 | 2.61E-01 | 8.34E-01 | 8.02E-01 | comfirm |
| CDKN2B | 1.09E-13 | 7.20E-05 | 1.16E-02 | 5.73E-01 | 2.74E-01 | comfirm |
| CFLAR | 8.80E-12 | 1.60E-01 | 9.90E-03 | #N/A | 9.86E-01 | comfirm |
| CBL | 7.08E-08 | 6.22E-01 | 7.30E-03 | 9.61E-01 | 7.23E-01 | comfirm |
| HSPA1A | 1.91E-07 | 2.58E-03 | 1.15E-02 | 7.99E-01 | 8.84E-01 | comfirm |
| NPC1 | 8.76E-270 | 9.00E-06 | 5.10E-02 | 9.87E-01 | 9.86E-01 | novel |
| GMPR | 7.22E-228 | 7.34E-01 | 3.75E-04 | 5.57E-01 | 9.49E-01 | novel |
| ZFAND4 | 4.37E-192 | 7.87E-01 | 3.26E-03 | 9.19E-01 | 9.86E-01 | novel |
| RGS22 | 1.25E-191 | 8.59E-02 | 3.00E-03 | #N/A | #N/A | novel |
| FGGY | 8.17E-187 | 1.68E-02 | 8.21E-03 | 9.60E-01 | 8.05E-01 | novel |
| NARS2 | 6.92E-174 | 2.08E-01 | 1.55E-03 | 9.89E-01 | 1.58E-01 | novel |
| HMGCLL1 | 1.32E-156 | 4.15E-02 | 3.18E-04 | #N/A | #N/A | novel |
| TM9SF3 | 1.13E-150 | 6.00E-05 | 7.21E-01 | #N/A | #N/A | novel |
| AP1G1 | 4.84E-149 | 8.58E-01 | 2.78E-03 | 9.85E-01 | 8.75E-01 | novel |
| LINC00536 | 6.39E-144 | 4.50E-03 | 3.55E-01 | #N/A | #N/A | novel |
| ZNF568 | 5.97E-128 | 8.51E-01 | 8.35E-03 | #N/A | #N/A | novel |
| SLC26A5 | 3.66E-115 | 1.69E-03 | 4.97E-01 | 8.39E-01 | 6.67E-01 | novel |
| CKAP5 | 2.55E-105 | 1.65E-01 | 1.12E-04 | #N/A | #N/A | novel |
| PSMD1 | 6.51E-101 | 9.99E-01 | 2.82E-03 | #N/A | #N/A | novel |
| PHACTR2 | 7.14E-89 | 5.08E-03 | 1.95E-01 | 7.79E-01 | 7.99E-01 | novel |
| PTPLB | 1.09E-81 | 5.31E-03 | 3.77E-02 | 8.26E-01 | 6.46E-01 | novel |
| R3HCC1L | 1.48E-81 | 1.46E-02 | 4.68E-03 | 8.21E-01 | 4.51E-01 | novel |
| ZBTB38 | 7.27E-77 | 8.91E-01 | 6.00E-06 | 8.53E-01 | 5.58E-02 | novel |
| TOM1L2 | 4.00E-74 | 4.26E-03 | 1.97E-02 | 3.25E-01 | 4.86E-01 | novel |
| LOC101927728 | 7.55E-70 | 2.50E-01 | 2.65E-03 | #N/A | #N/A | novel |
| EML6 | 6.18E-69 | 7.59E-01 | 4.44E-03 | 8.87E-01 | 2.70E-01 | novel |
| FAM179B | 2.06E-68 | 6.06E-03 | 1.19E-01 | #N/A | #N/A | novel |
| GGNBP2 | 6.17E-68 | 3.92E-02 | 1.00E-06 | 6.85E-01 | 3.15E-02 | novel |
| PTPRJ | 1.98E-64 | 1.29E-01 | 2.90E-03 | 9.07E-01 | 8.34E-01 | novel |
| KCNG3 | 2.58E-58 | 5.65E-03 | 4.83E-01 | #N/A | #N/A | novel |
| HSF2BP | 4.84E-58 | 1.01E-02 | 4.21E-03 | #N/A | #N/A | novel |
| SNX6 | 1.30E-57 | 6.09E-01 | 6.48E-03 | 9.74E-01 | 5.78E-01 | novel |
| IGF2BP1 | 2.41E-54 | 4.97E-04 | 5.06E-02 | #N/A | #N/A | novel |
| ZNF43 | 1.79E-53 | 4.92E-03 | 1.80E-01 | 9.31E-01 | 6.38E-01 | novel |
| C11orf49 | 1.34E-52 | 2.20E-01 | 3.34E-04 | #N/A | #N/A | novel |
| METAP1 | 2.40E-52 | 2.91E-01 | 3.15E-03 | #N/A | #N/A | novel |
| AGBL2 | 5.86E-52 | 1.47E-01 | 1.80E-05 | #N/A | #N/A | novel |
| NEK5 | 2.95E-51 | 8.62E-03 | 3.05E-01 | #N/A | #N/A | novel |
| MIR548AI | 5.10E-51 | 1.04E-04 | 1.71E-01 | #N/A | #N/A | novel |
| DCDC5 | 1.06E-49 | 5.28E-02 | 1.77E-03 | #N/A | #N/A | novel |
| ARMC9 | 2.35E-49 | 9.51E-01 | 2.18E-03 | 8.48E-01 | 9.22E-01 | novel |
| ATP5J2 | 5.82E-46 | 4.93E-03 | 9.97E-01 | 9.74E-01 | 7.38E-01 | novel |
| TNIK | 2.02E-45 | 8.49E-02 | 6.52E-03 | #N/A | #N/A | novel |
| FBXO42 | 3.60E-44 | 5.28E-02 | 8.32E-03 | 8.53E-01 | 3.79E-01 | novel |
| CYTH1 | 1.64E-43 | 1.38E-03 | 3.74E-01 | #N/A | #N/A | novel |
| ASAP3 | 1.79E-41 | 2.11E-01 | 3.94E-04 | #N/A | #N/A | novel |
| ARHGEF3 | 8.23E-38 | 8.69E-03 | 7.79E-02 | 9.28E-01 | 4.18E-01 | novel |
| PIGU | 2.32E-37 | 4.26E-02 | 4.25E-03 | 6.28E-01 | 6.60E-01 | novel |
| L3MBTL4 | 4.53E-37 | 1.27E-01 | 8.53E-03 | #N/A | #N/A | novel |
| SLC10A7 | 1.91E-33 | 1.20E-01 | 1.31E-03 | 9.67E-01 | 9.69E-01 | novel |
| TAF4 | 1.09E-32 | 4.42E-01 | 1.59E-03 | 9.31E-01 | 8.61E-01 | novel |
| MBOAT2 | 2.91E-31 | 2.90E-01 | 2.72E-03 | 7.67E-01 | 8.89E-01 | novel |
| FANCM | 1.69E-30 | 5.73E-03 | 5.22E-02 | 3.77E-01 | 7.45E-01 | novel |
| HECW1 | 4.85E-30 | 3.11E-01 | 6.32E-03 | #N/A | #N/A | novel |
| INPP5B | 1.24E-28 | 2.89E-03 | 6.15E-02 | 1.98E-01 | 5.83E-01 | novel |
| BTBD10 | 4.37E-28 | 2.38E-03 | 6.00E-01 | 8.94E-01 | 9.30E-01 | novel |
| KPNA4 | 1.90E-27 | 2.23E-03 | 3.15E-01 | #N/A | #N/A | novel |
| TMEM241 | 3.72E-26 | 8.00E-03 | 3.99E-01 | 7.90E-01 | 7.96E-01 | novel |
| RASA2 | 1.58E-25 | 5.19E-01 | 1.49E-04 | #N/A | #N/A | novel |
| L3MBTL3 | 3.29E-25 | 7.37E-02 | 1.23E-04 | 5.45E-01 | 1.47E-02 | novel |
| GRB10 | 5.44E-25 | 2.89E-03 | 3.07E-01 | 1.56E-01 | 5.83E-01 | novel |
| GABRA2 | 1.19E-22 | 6.28E-03 | 7.35E-01 | #N/A | #N/A | novel |
| SEC23B | 3.68E-22 | 4.16E-03 | 5.02E-02 | 3.03E-01 | 5.37E-01 | novel |
| HNRNPM | 3.72E-22 | 1.84E-03 | 8.89E-01 | 6.24E-01 | 8.69E-01 | novel |
| NAALAD2 | 4.00E-22 | 5.15E-03 | 4.30E-01 | 1.98E-01 | 9.45E-01 | novel |
| ECHDC3 | 5.29E-22 | 5.96E-01 | 8.28E-03 | 8.51E-01 | 5.57E-01 | novel |
| CEP89 | 4.87E-20 | 3.10E-01 | 5.32E-04 | #N/A | #N/A | novel |
| HIGD1C | 1.36E-18 | 2.87E-03 | 7.78E-01 | #N/A | #N/A | novel |
| GADL1 | 4.62E-18 | 1.43E-02 | 1.73E-03 | #N/A | #N/A | novel |
| HM13 | 6.72E-17 | 1.00E-02 | 3.62E-01 | 3.94E-01 | 9.64E-01 | novel |
| ABHD17C | 6.97E-17 | 8.59E-04 | 7.09E-01 | 8.36E-01 | 9.02E-01 | novel |
| CPNE3 | 3.81E-16 | 4.72E-01 | 9.20E-03 | 8.65E-01 | 9.89E-01 | novel |
| TFCP2 | 2.33E-15 | 6.82E-03 | 4.53E-01 | #N/A | #N/A | novel |
| SLC44A1 | 2.76E-15 | 3.54E-01 | 5.76E-03 | #N/A | #N/A | novel |
| CHRM3 | 4.58E-15 | 6.40E-03 | 8.34E-01 | #N/A | #N/A | novel |
| ST20 | 7.82E-15 | 7.07E-04 | 4.21E-01 | 8.01E-01 | 8.95E-01 | novel |
| DZANK1 | 1.14E-14 | 3.74E-02 | 4.08E-03 | 9.55E-01 | 4.21E-01 | novel |
| ZNF805 | 7.43E-14 | 4.05E-03 | 9.28E-01 | #N/A | #N/A | novel |
| LINC01111 | 7.49E-14 | 7.49E-02 | 6.91E-03 | #N/A | #N/A | novel |
| ASXL3 | 8.73E-14 | 3.70E-03 | 4.73E-01 | #N/A | #N/A | novel |
| TNRC6A | 2.39E-13 | 4.58E-03 | 5.49E-01 | 9.74E-01 | 6.67E-01 | novel |
| LRPAP1 | 5.31E-13 | 5.03E-01 | 5.22E-03 | 8.53E-01 | 3.08E-01 | novel |
| UBE2E1 | 9.33E-12 | 1.41E-03 | 5.43E-01 | #N/A | #N/A | novel |
| POM121C | 1.50E-11 | 7.09E-04 | 5.68E-01 | 4.55E-02 | 8.27E-01 | novel |
| SSH1 | 2.78E-11 | 3.61E-01 | 5.45E-03 | 8.66E-01 | 6.69E-01 | novel |
| ALMS1P | 4.63E-11 | 1.56E-01 | 1.13E-04 | 8.20E-01 | 7.03E-04 | novel |
| PAIP2B | 3.15E-10 | 4.17E-01 | 7.03E-03 | #N/A | #N/A | novel |
| LINC00607 | 5.99E-10 | 4.47E-03 | 4.91E-01 | #N/A | #N/A | novel |
| ANO5 | 6.06E-10 | 2.32E-01 | 7.15E-03 | 9.91E-01 | 5.83E-01 | novel |
| SYTL3 | 6.88E-10 | 6.30E-01 | 4.57E-03 | 9.21E-01 | 8.72E-01 | novel |
| CCDC158 | 3.78E-09 | 2.10E-01 | 1.00E-06 | #N/A | #N/A | novel |
| IFT74 | 9.46E-09 | 3.72E-01 | 3.25E-03 | 8.48E-01 | 7.56E-01 | novel |
| ACAD10 | 1.04E-08 | 1.73E-03 | 8.89E-02 | #N/A | #N/A | novel |
| CRTC1 | 1.21E-08 | 7.44E-03 | 9.50E-03 | #N/A | #N/A | novel |
| PPAP2B | 2.06E-08 | 1.68E-04 | 2.25E-01 | 5.53E-01 | 9.54E-01 | novel |
| ACVR1C | 3.07E-08 | 6.12E-01 | 1.00E-02 | 9.96E-01 | 7.99E-01 | novel |
| GPR149 | 5.16E-08 | 1.49E-01 | 7.13E-04 | #N/A | #N/A | novel |
| ZFP14 | 6.02E-08 | 4.23E-02 | 5.63E-03 | #N/A | #N/A | novel |
| FAM120B | 8.13E-08 | 8.79E-03 | 3.88E-02 | 2.71E-01 | 6.64E-01 | novel |
| FAM53B | 9.97E-08 | 7.01E-01 | 3.04E-03 | 8.63E-01 | 9.67E-01 | novel |
| HECTD4 | 1.90E-07 | 4.79E-03 | 6.65E-01 | 8.52E-01 | 9.89E-01 | novel |
| MIR100HG | 2.27E-07 | 5.62E-01 | 2.38E-03 | #N/A | #N/A | novel |
| TTLL6 | 4.45E-07 | 9.80E-03 | 4.13E-01 | #N/A | #N/A | novel |
| STYXL1 | 4.69E-07 | 3.80E-01 | 1.75E-03 | 9.58E-01 | 6.42E-01 | novel |
| RNF38 | 6.29E-07 | 4.58E-03 | 5.31E-01 | #N/A | #N/A | novel |
| ATP6V1G2 | 1.40E-297 | 9.39E-02 | 1.31E-04 | 6.63E-01 | 6.64E-01 | potential |
| MARK3 | 2.17E-281 | 8.90E-03 | 2.22E-02 | 9.62E-01 | 2.34E-01 | potential |
| MADD | 1.82E-272 | 3.06E-01 | 2.45E-04 | 8.98E-01 | 7.88E-01 | potential |
| RIN3 | 5.06E-270 | 4.65E-03 | 2.77E-01 | #N/A | #N/A | potential |
| WWP2 | 1.03E-210 | 5.22E-03 | 8.19E-02 | 9.67E-01 | 7.62E-01 | potential |
| MYO1D | 8.18E-189 | 1.86E-01 | 1.17E-03 | 8.52E-01 | 9.87E-01 | potential |
| STRN | 7.46E-160 | 6.89E-04 | 9.22E-01 | 6.40E-01 | 9.99E-01 | potential |
| ATF1 | 1.29E-141 | 1.24E-03 | 6.11E-01 | 1.52E-01 | 9.60E-01 | potential |
| CDON | 1.40E-124 | 6.88E-03 | 5.05E-02 | #N/A | #N/A | potential |
| CHMP3 | 1.02E-113 | 6.45E-03 | 1.21E-01 | 1.24E-01 | 6.64E-01 | potential |
| PLSCR4 | 5.93E-103 | 4.51E-03 | 6.71E-01 | #N/A | #N/A | potential |
| NUP37 | 2.26E-78 | 4.56E-03 | 9.00E-01 | #N/A | #N/A | potential |
| LMCD1 | 5.22E-65 | 8.43E-03 | 9.24E-01 | 8.65E-01 | 9.78E-01 | potential |
| GNB1 | 5.23E-56 | 3.18E-01 | 5.32E-03 | #N/A | #N/A | potential |
| SORT1 | 7.81E-52 | 1.49E-01 | 1.80E-03 | #N/A | #N/A | potential |
| ADAM10 | 3.33E-50 | 5.02E-03 | 3.72E-01 | 8.52E-01 | 9.46E-01 | potential |
| PLXND1 | 2.37E-36 | 4.85E-01 | 2.84E-03 | 8.97E-01 | 4.85E-01 | potential |
| FAM47E | 2.69E-34 | 3.74E-01 | 1.00E-06 | #N/A | #N/A | potential |
| COL4A4 | 7.89E-33 | 2.71E-03 | 3.82E-01 | #N/A | #N/A | potential |
| HS6ST1 | 8.11E-32 | 2.31E-01 | 9.90E-03 | #N/A | #N/A | potential |
| CC2D2A | 3.69E-31 | 5.56E-03 | 8.36E-01 | 7.31E-01 | 8.29E-01 | potential |
| FGF5 | 1.15E-26 | 4.31E-01 | 1.39E-03 | #N/A | #N/A | potential |
| PDIA6 | 2.78E-26 | 5.57E-02 | 9.59E-04 | 8.67E-01 | 2.76E-01 | potential |
| TCF7L1 | 2.87E-23 | 8.22E-01 | 1.36E-03 | 7.28E-01 | 1.58E-01 | potential |
| KCNK10 | 4.34E-23 | 7.07E-01 | 9.00E-03 | #N/A | #N/A | potential |
| VTI1A | 1.00E-22 | 7.65E-03 | 3.51E-02 | 8.66E-01 | 9.30E-01 | potential |
| PEX7 | 2.26E-22 | 8.30E-01 | 4.41E-03 | 8.93E-01 | 9.89E-01 | potential |
| RERG | 2.35E-21 | 9.09E-01 | 5.00E-06 | #N/A | #N/A | potential |
| KNG1 | 1.18E-20 | 1.71E-01 | 1.30E-04 | #N/A | #N/A | potential |
| KCNG2 | 1.21E-20 | 3.81E-01 | 1.00E-02 | 7.87E-01 | 9.89E-01 | potential |
| ATXN7 | 1.70E-18 | 1.53E-01 | 4.90E-05 | 8.98E-01 | 4.07E-01 | potential |
| BCAS3 | 2.27E-17 | 1.68E-01 | 1.24E-03 | #N/A | #N/A | potential |
| TINAG | 6.59E-17 | 3.60E-01 | 1.60E-05 | #N/A | #N/A | potential |
| LAMC2 | 2.38E-13 | 6.84E-01 | 9.90E-03 | #N/A | #N/A | potential |
| CASZ1 | 8.78E-11 | 1.10E-01 | 1.92E-04 | 8.62E-01 | 9.82E-01 | potential |
| GAB2 | 6.41E-10 | 8.56E-01 | 1.80E-05 | #N/A | 4.61E-02 | potential |
| TFCP2L1 | 1.02E-09 | 4.06E-01 | 4.00E-06 | 6.81E-01 | 7.88E-01 | potential |
| ATXN2 | 1.88E-09 | 1.23E-04 | 3.53E-02 | #N/A | 9.53E-01 | potential |
| GATM | 3.75E-09 | 3.04E-01 | 1.00E-06 | 8.38E-01 | 7.77E-07 | potential |
| BCL9 | 1.11E-08 | 7.86E-03 | 8.29E-02 | #N/A | #N/A | potential |
| NCOA6 | 1.30E-07 | 2.43E-01 | 7.56E-03 | #N/A | #N/A | potential |
| LIPE | 2.88E-07 | 2.03E-03 | 6.76E-01 | 3.23E-01 | 9.69E-01 | potential |
| LAMA5 | 5.90E-07 | 6.96E-01 | 5.09E-03 | 8.83E-01 | 8.58E-01 | potential |
