# Supplementary material for: Exploring the Pleiotropic Genes and Therapeutic Targets Associated with Heart Failure and Chronic Kidney Disease by Integrating metaCCA and SGLT2 Inhibitors' Target Prediction
Source: Biomed Res Int. 2021 Sep 8;2021:4229194. doi: 10.1155/2021/4229194 (PMC8443964; doi:10.1155/2021/4229194)
Supplement: Supplementary 4 — Table S4: GO BP enrichment. [file 4229194.f4.docx]

| ID | Description | GeneRatio | BgRatio | pvalue | p.adjust | qvalue | Count |
| --- | --- | --- | --- | --- | --- | --- | --- |
| GO:0009266 | response to temperature stimulus | 17/193 | 269/21081 | 4.98E-10 | 1.23E-06 | 1.04E-06 | 17 |
| GO:0090063 | positive regulation of microtubule nucleation | 6/193 | 13/21081 | 8.86E-10 | 1.23E-06 | 1.04E-06 | 6 |
| GO:0010968 | regulation of microtubule nucleation | 6/193 | 15/21081 | 2.55E-09 | 2.35E-06 | 1.99E-06 | 6 |
| GO:0009408 | response to heat | 14/193 | 201/21081 | 5.15E-09 | 3.57E-06 | 3.02E-06 | 14 |
| GO:0090383 | phagosome acidification | 7/193 | 35/21081 | 2.62E-08 | 1.25E-05 | 1.06E-05 | 7 |
| GO:0070434 | positive regulation of nucleotide-binding oligomerization domain containing 2 signaling pathway | 5/193 | 11/21081 | 2.70E-08 | 1.25E-05 | 1.06E-05 | 5 |
| GO:0032075 | positive regulation of nuclease activity | 5/193 | 12/21081 | 4.59E-08 | 1.59E-05 | 1.35E-05 | 5 |
| GO:0070426 | positive regulation of nucleotide-binding oligomerization domain containing signaling pathway | 5/193 | 12/21081 | 4.59E-08 | 1.59E-05 | 1.35E-05 | 5 |
| GO:0010286 | heat acclimation | 5/193 | 13/21081 | 7.41E-08 | 1.87E-05 | 1.58E-05 | 5 |
| GO:0060700 | regulation of ribonuclease activity | 5/193 | 13/21081 | 7.41E-08 | 1.87E-05 | 1.58E-05 | 5 |
| GO:0070370 | cellular heat acclimation | 5/193 | 13/21081 | 7.41E-08 | 1.87E-05 | 1.58E-05 | 5 |
| GO:0031116 | positive regulation of microtubule polymerization | 7/193 | 41/21081 | 8.35E-08 | 1.93E-05 | 1.64E-05 | 7 |
| GO:0070432 | regulation of nucleotide-binding oligomerization domain containing 2 signaling pathway | 5/193 | 14/21081 | 1.14E-07 | 2.33E-05 | 1.98E-05 | 5 |
| GO:0033572 | transferrin transport | 7/193 | 43/21081 | 1.18E-07 | 2.33E-05 | 1.98E-05 | 7 |
| GO:0032868 | response to insulin | 15/193 | 303/21081 | 1.38E-07 | 2.56E-05 | 2.17E-05 | 15 |
| GO:0031112 | positive regulation of microtubule polymerization or depolymerization | 7/193 | 45/21081 | 1.63E-07 | 2.83E-05 | 2.40E-05 | 7 |
| GO:0007041 | lysosomal transport | 10/193 | 123/21081 | 2.01E-07 | 3.27E-05 | 2.77E-05 | 10 |
| GO:1900034 | regulation of cellular response to heat | 9/193 | 96/21081 | 2.45E-07 | 3.59E-05 | 3.04E-05 | 9 |
| GO:1901029 | negative regulation of mitochondrial outer membrane permeabilization involved in apoptotic signaling pathway | 5/193 | 16/21081 | 2.46E-07 | 3.59E-05 | 3.04E-05 | 5 |
| GO:0051131 | chaperone-mediated protein complex assembly | 6/193 | 30/21081 | 2.69E-07 | 3.74E-05 | 3.16E-05 | 6 |
| GO:0097201 | negative regulation of transcription from RNA polymerase II promoter in response to stress | 5/193 | 17/21081 | 3.46E-07 | 4.56E-05 | 3.87E-05 | 5 |
| GO:0007034 | vacuolar transport | 11/193 | 166/21081 | 3.91E-07 | 4.93E-05 | 4.17E-05 | 11 |
| GO:0032869 | cellular response to insulin stimulus | 13/193 | 245/21081 | 4.42E-07 | 5.33E-05 | 4.52E-05 | 13 |
| GO:0070424 | regulation of nucleotide-binding oligomerization domain containing signaling pathway | 5/193 | 18/21081 | 4.75E-07 | 5.49E-05 | 4.65E-05 | 5 |
| GO:1903265 | positive regulation of tumor necrosis factor-mediated signaling pathway | 5/193 | 19/21081 | 6.40E-07 | 7.10E-05 | 6.01E-05 | 5 |
| GO:0090382 | phagosome maturation | 7/193 | 56/21081 | 7.67E-07 | 8.10E-05 | 6.86E-05 | 7 |
| GO:0042026 | protein refolding | 6/193 | 36/21081 | 8.44E-07 | 8.10E-05 | 6.86E-05 | 6 |
| GO:0032815 | negative regulation of natural killer cell activation | 5/193 | 20/21081 | 8.47E-07 | 8.10E-05 | 6.86E-05 | 5 |
| GO:0090084 | negative regulation of inclusion body assembly | 5/193 | 20/21081 | 8.47E-07 | 8.10E-05 | 6.86E-05 | 5 |
| GO:0071375 | cellular response to peptide hormone stimulus | 15/193 | 359/21081 | 1.19E-06 | 0.00011 | 9.32E-05 | 15 |
| GO:0070431 | nucleotide-binding oligomerization domain containing 2 signaling pathway | 5/193 | 22/21081 | 1.42E-06 | 0.000127 | 0.000107 | 5 |
| GO:0008286 | insulin receptor signaling pathway | 10/193 | 153/21081 | 1.51E-06 | 0.000131 | 0.000111 | 10 |
| GO:0006826 | iron ion transport | 8/193 | 89/21081 | 1.60E-06 | 0.000134 | 0.000114 | 8 |
| GO:0031113 | regulation of microtubule polymerization | 7/193 | 63/21081 | 1.73E-06 | 0.000137 | 0.000116 | 7 |
| GO:0051452 | intracellular pH reduction | 7/193 | 63/21081 | 1.73E-06 | 0.000137 | 0.000116 | 7 |
| GO:0007020 | microtubule nucleation | 6/193 | 41/21081 | 1.88E-06 | 0.000145 | 0.000122 | 6 |
| GO:0043434 | response to peptide hormone | 17/193 | 478/21081 | 2.08E-06 | 0.000156 | 0.000132 | 17 |
| GO:0051085 | chaperone cofactor-dependent protein refolding | 6/193 | 43/21081 | 2.51E-06 | 0.00018 | 0.000153 | 6 |
| GO:0045851 | pH reduction | 7/193 | 67/21081 | 2.64E-06 | 0.00018 | 0.000153 | 7 |
| GO:1901653 | cellular response to peptide | 16/193 | 434/21081 | 2.65E-06 | 0.00018 | 0.000153 | 16 |
| GO:0090083 | regulation of inclusion body assembly | 5/193 | 25/21081 | 2.80E-06 | 0.00018 | 0.000153 | 5 |
| GO:1901673 | regulation of mitotic spindle assembly | 5/193 | 25/21081 | 2.80E-06 | 0.00018 | 0.000153 | 5 |
| GO:1902236 | negative regulation of endoplasmic reticulum stress-induced intrinsic apoptotic signaling pathway | 5/193 | 25/21081 | 2.80E-06 | 0.00018 | 0.000153 | 5 |
| GO:0032069 | regulation of nuclease activity | 5/193 | 26/21081 | 3.44E-06 | 0.000217 | 0.000183 | 5 |
| GO:0031110 | regulation of microtubule polymerization or depolymerization | 8/193 | 99/21081 | 3.59E-06 | 0.000221 | 0.000187 | 8 |
| GO:1903844 | regulation of cellular response to transforming growth factor beta stimulus | 9/193 | 136/21081 | 4.61E-06 | 0.000278 | 0.000236 | 9 |
| GO:0051084 | 'de novo' posttranslational protein folding | 6/193 | 48/21081 | 4.86E-06 | 0.000286 | 0.000243 | 6 |
| GO:0032814 | regulation of natural killer cell activation | 6/193 | 51/21081 | 6.97E-06 | 0.000402 | 0.000341 | 6 |
| GO:0006458 | 'de novo' protein folding | 6/193 | 52/21081 | 7.81E-06 | 0.000442 | 0.000375 | 6 |
| GO:0000041 | transition metal ion transport | 9/193 | 146/21081 | 8.23E-06 | 0.000449 | 0.00038 | 9 |
| GO:0052126 | movement in host environment | 11/193 | 227/21081 | 8.27E-06 | 0.000449 | 0.00038 | 11 |
| GO:0002418 | immune response to tumor cell | 5/193 | 31/21081 | 8.55E-06 | 0.000449 | 0.00038 | 5 |
| GO:0034605 | cellular response to heat | 9/193 | 147/21081 | 8.70E-06 | 0.000449 | 0.00038 | 9 |
| GO:0010803 | regulation of tumor necrosis factor-mediated signaling pathway | 7/193 | 80/21081 | 8.73E-06 | 0.000449 | 0.00038 | 7 |
| GO:0051701 | interaction with host | 12/193 | 281/21081 | 1.15E-05 | 0.000581 | 0.000492 | 12 |
| GO:0090169 | regulation of spindle assembly | 5/193 | 33/21081 | 1.18E-05 | 0.000583 | 0.000494 | 5 |
| GO:0061013 | regulation of mRNA catabolic process | 11/193 | 237/21081 | 1.24E-05 | 0.000603 | 0.000511 | 11 |
| GO:0010506 | regulation of autophagy | 14/193 | 385/21081 | 1.33E-05 | 0.000636 | 0.000539 | 14 |
| GO:0016241 | regulation of macroautophagy | 10/193 | 197/21081 | 1.43E-05 | 0.000663 | 0.000562 | 10 |
| GO:0044409 | entry into host | 10/193 | 197/21081 | 1.43E-05 | 0.000663 | 0.000562 | 10 |
| GO:0046629 | gamma-delta T cell activation | 5/193 | 35/21081 | 1.59E-05 | 0.000721 | 0.000611 | 5 |
| GO:0002347 | response to tumor cell | 5/193 | 37/21081 | 2.10E-05 | 0.000924 | 0.000782 | 5 |
| GO:1902235 | regulation of endoplasmic reticulum stress-induced intrinsic apoptotic signaling pathway | 5/193 | 37/21081 | 2.10E-05 | 0.000924 | 0.000782 | 5 |
| GO:0046785 | microtubule polymerization | 7/193 | 93/21081 | 2.36E-05 | 0.001021 | 0.000865 | 7 |
| GO:1903845 | negative regulation of cellular response to transforming growth factor beta stimulus | 7/193 | 95/21081 | 2.71E-05 | 0.001155 | 0.000978 | 7 |
| GO:0070507 | regulation of microtubule cytoskeleton organization | 10/193 | 215/21081 | 3.04E-05 | 0.001279 | 0.001083 | 10 |
| GO:0051453 | regulation of intracellular pH | 7/193 | 97/21081 | 3.10E-05 | 0.001283 | 0.001087 | 7 |
| GO:0017015 | regulation of transforming growth factor beta receptor signaling pathway | 8/193 | 134/21081 | 3.33E-05 | 0.001351 | 0.001145 | 8 |
| GO:0071560 | cellular response to transforming growth factor beta stimulus | 11/193 | 265/21081 | 3.48E-05 | 0.001351 | 0.001145 | 11 |
| GO:0019835 | cytolysis | 5/193 | 41/21081 | 3.50E-05 | 0.001351 | 0.001145 | 5 |
| GO:0070841 | inclusion body assembly | 5/193 | 41/21081 | 3.50E-05 | 0.001351 | 0.001145 | 5 |
| GO:0031109 | microtubule polymerization or depolymerization | 8/193 | 135/21081 | 3.51E-05 | 0.001351 | 0.001145 | 8 |
| GO:0032886 | regulation of microtubule-based process | 11/193 | 267/21081 | 3.73E-05 | 0.001416 | 0.0012 | 11 |
| GO:0060236 | regulation of mitotic spindle organization | 5/193 | 42/21081 | 3.95E-05 | 0.001479 | 0.001253 | 5 |
| GO:0043618 | regulation of transcription from RNA polymerase II promoter in response to stress | 8/193 | 138/21081 | 4.10E-05 | 0.001518 | 0.001286 | 8 |
| GO:0071559 | response to transforming growth factor beta | 11/193 | 271/21081 | 4.27E-05 | 0.001557 | 0.001319 | 11 |
| GO:0033120 | positive regulation of RNA splicing | 5/193 | 43/21081 | 4.43E-05 | 0.001576 | 0.001335 | 5 |
| GO:0045648 | positive regulation of erythrocyte differentiation | 5/193 | 43/21081 | 4.43E-05 | 0.001576 | 0.001335 | 5 |
| GO:0010823 | negative regulation of mitochondrion organization | 6/193 | 71/21081 | 4.76E-05 | 0.001671 | 0.001416 | 6 |
| GO:0030641 | regulation of cellular pH | 7/193 | 104/21081 | 4.86E-05 | 0.001683 | 0.001426 | 7 |
| GO:0061077 | chaperone-mediated protein folding | 6/193 | 72/21081 | 5.15E-05 | 0.001764 | 0.001495 | 6 |
| GO:0043620 | regulation of DNA-templated transcription in response to stress | 8/193 | 145/21081 | 5.83E-05 | 0.001973 | 0.001672 | 8 |
| GO:0070423 | nucleotide-binding oligomerization domain containing signaling pathway | 5/193 | 46/21081 | 6.17E-05 | 0.002062 | 0.001747 | 5 |
| GO:0035872 | nucleotide-binding domain, leucine rich repeat containing receptor signaling pathway | 5/193 | 47/21081 | 6.86E-05 | 0.002237 | 0.001895 | 5 |
| GO:0090224 | regulation of spindle organization | 5/193 | 47/21081 | 6.86E-05 | 0.002237 | 0.001895 | 5 |
| GO:0006885 | regulation of pH | 7/193 | 114/21081 | 8.71E-05 | 0.002809 | 0.00238 | 7 |
| GO:0006110 | regulation of glycolytic process | 6/193 | 81/21081 | 1.00E-04 | 0.003176 | 0.002691 | 6 |
| GO:1901099 | negative regulation of signal transduction in absence of ligand | 5/193 | 51/21081 | 0.000102 | 0.003176 | 0.002691 | 5 |
| GO:2001240 | negative regulation of extrinsic apoptotic signaling pathway in absence of ligand | 5/193 | 51/21081 | 0.000102 | 0.003176 | 0.002691 | 5 |
| GO:0032273 | positive regulation of protein polymerization | 8/193 | 159/21081 | 0.000111 | 0.003431 | 0.002907 | 8 |
| GO:0014889 | muscle atrophy | 3/193 | 11/21081 | 0.000118 | 0.003599 | 0.003049 | 3 |
| GO:0030004 | cellular monovalent inorganic cation homeostasis | 7/193 | 125/21081 | 0.000155 | 0.004676 | 0.003962 | 7 |
| GO:0043488 | regulation of mRNA stability | 9/193 | 213/21081 | 0.000158 | 0.004712 | 0.003992 | 9 |
| GO:1901028 | regulation of mitochondrial outer membrane permeabilization involved in apoptotic signaling pathway | 5/193 | 56/21081 | 0.00016 | 0.004712 | 0.003992 | 5 |
| GO:0007179 | transforming growth factor beta receptor signaling pathway | 9/193 | 214/21081 | 0.000164 | 0.00478 | 0.00405 | 9 |
| GO:0050821 | protein stabilization | 9/193 | 220/21081 | 0.000201 | 0.00578 | 0.004897 | 9 |
| GO:0043470 | regulation of carbohydrate catabolic process | 6/193 | 92/21081 | 0.000202 | 0.00578 | 0.004897 | 6 |
| GO:0030512 | negative regulation of transforming growth factor beta receptor signaling pathway | 6/193 | 93/21081 | 0.000215 | 0.006069 | 0.005142 | 6 |
| GO:0031331 | positive regulation of cellular catabolic process | 13/193 | 441/21081 | 0.000224 | 0.006277 | 0.005318 | 13 |
| GO:0061099 | negative regulation of protein tyrosine kinase activity | 4/193 | 33/21081 | 0.000226 | 0.006277 | 0.005318 | 4 |
| GO:0062208 | positive regulation of pattern recognition receptor signaling pathway | 5/193 | 61/21081 | 0.00024 | 0.006582 | 0.005577 | 5 |
| GO:0043487 | regulation of RNA stability | 9/193 | 227/21081 | 0.000254 | 0.006902 | 0.005848 | 9 |
| GO:0045646 | regulation of erythrocyte differentiation | 5/193 | 62/21081 | 0.000259 | 0.006902 | 0.005848 | 5 |
| GO:1903573 | negative regulation of response to endoplasmic reticulum stress | 5/193 | 62/21081 | 0.000259 | 0.006902 | 0.005848 | 5 |
| GO:0033209 | tumor necrosis factor-mediated signaling pathway | 9/193 | 228/21081 | 0.000262 | 0.006931 | 0.005873 | 9 |
| GO:0010639 | negative regulation of organelle organization | 13/193 | 449/21081 | 0.000267 | 0.006973 | 0.005908 | 13 |
| GO:0051091 | positive regulation of DNA-binding transcription factor activity | 11/193 | 335/21081 | 0.000276 | 0.00715 | 0.006058 | 11 |
| GO:2001239 | regulation of extrinsic apoptotic signaling pathway in absence of ligand | 5/193 | 63/21081 | 0.000279 | 0.007166 | 0.006072 | 5 |
| GO:0071482 | cellular response to light stimulus | 7/193 | 138/21081 | 0.000285 | 0.00726 | 0.006151 | 7 |
| GO:0051258 | protein polymerization | 11/193 | 337/21081 | 0.00029 | 0.007317 | 0.006199 | 11 |
| GO:0046718 | viral entry into host cell | 8/193 | 184/21081 | 0.000302 | 0.007556 | 0.006402 | 8 |
| GO:0016236 | macroautophagy | 11/193 | 340/21081 | 0.000313 | 0.007748 | 0.006565 | 11 |
| GO:0046034 | ATP metabolic process | 11/193 | 342/21081 | 0.000329 | 0.008071 | 0.006838 | 11 |
| GO:0001961 | positive regulation of cytokine-mediated signaling pathway | 5/193 | 67/21081 | 0.000372 | 0.008978 | 0.007607 | 5 |
| GO:0097345 | mitochondrial outer membrane permeabilization | 5/193 | 67/21081 | 0.000372 | 0.008978 | 0.007607 | 5 |
| GO:0042391 | regulation of membrane potential | 13/193 | 473/21081 | 0.000438 | 0.010459 | 0.008862 | 13 |
| GO:0001659 | temperature homeostasis | 8/193 | 195/21081 | 0.000446 | 0.010569 | 0.008955 | 8 |
| GO:0030218 | erythrocyte differentiation | 7/193 | 149/21081 | 0.000454 | 0.010668 | 0.009039 | 7 |
| GO:0030522 | intracellular receptor signaling pathway | 10/193 | 302/21081 | 0.000489 | 0.011387 | 0.009648 | 10 |
| GO:0120162 | positive regulation of cold-induced thermogenesis | 6/193 | 109/21081 | 0.000505 | 0.011672 | 0.009889 | 6 |
| GO:0032757 | positive regulation of interleukin-8 production | 5/193 | 72/21081 | 0.00052 | 0.011915 | 0.010095 | 5 |
| GO:1902110 | positive regulation of mitochondrial membrane permeability involved in apoptotic process | 5/193 | 73/21081 | 0.000554 | 0.012593 | 0.01067 | 5 |
| GO:0070059 | intrinsic apoptotic signaling pathway in response to endoplasmic reticulum stress | 5/193 | 74/21081 | 0.00059 | 0.013245 | 0.011222 | 5 |
| GO:0006457 | protein folding | 9/193 | 255/21081 | 0.000592 | 0.013245 | 0.011222 | 9 |
| GO:0032271 | regulation of protein polymerization | 9/193 | 256/21081 | 0.000609 | 0.013512 | 0.011448 | 9 |
| GO:0060760 | positive regulation of response to cytokine stimulus | 5/193 | 75/21081 | 0.000627 | 0.013697 | 0.011605 | 5 |
| GO:1902686 | mitochondrial outer membrane permeabilization involved in programmed cell death | 5/193 | 75/21081 | 0.000627 | 0.013697 | 0.011605 | 5 |
| GO:0097191 | extrinsic apoptotic signaling pathway | 9/193 | 258/21081 | 0.000644 | 0.013848 | 0.011733 | 9 |
| GO:0034101 | erythrocyte homeostasis | 7/193 | 158/21081 | 0.000644 | 0.013848 | 0.011733 | 7 |
| GO:1902170 | cellular response to reactive nitrogen species | 3/193 | 19/21081 | 0.000657 | 0.013992 | 0.011855 | 3 |
| GO:0006986 | response to unfolded protein | 8/193 | 207/21081 | 0.000661 | 0.013992 | 0.011855 | 8 |
| GO:0045732 | positive regulation of protein catabolic process | 9/193 | 261/21081 | 0.000699 | 0.014688 | 0.012445 | 9 |
| GO:0035794 | positive regulation of mitochondrial membrane permeability | 5/193 | 77/21081 | 0.000708 | 0.014754 | 0.0125 | 5 |
| GO:0090307 | mitotic spindle assembly | 5/193 | 78/21081 | 0.000751 | 0.015417 | 0.013062 | 5 |
| GO:1902108 | regulation of mitochondrial membrane permeability involved in apoptotic process | 5/193 | 78/21081 | 0.000751 | 0.015417 | 0.013062 | 5 |
| GO:0071871 | response to epinephrine | 3/193 | 20/21081 | 0.000768 | 0.015656 | 0.013265 | 3 |
| GO:0006096 | glycolytic process | 6/193 | 119/21081 | 0.000803 | 0.016249 | 0.013767 | 6 |
| GO:0016052 | carbohydrate catabolic process | 8/193 | 214/21081 | 0.000821 | 0.016328 | 0.013834 | 8 |
| GO:0098926 | postsynaptic signal transduction | 4/193 | 46/21081 | 0.000823 | 0.016328 | 0.013834 | 4 |
| GO:0007178 | transmembrane receptor protein serine/threonine kinase signaling pathway | 11/193 | 382/21081 | 0.000824 | 0.016328 | 0.013834 | 11 |
| GO:0043467 | regulation of generation of precursor metabolites and energy | 7/193 | 165/21081 | 0.000832 | 0.016331 | 0.013837 | 7 |
| GO:0006757 | ATP generation from ADP | 6/193 | 120/21081 | 0.000839 | 0.016331 | 0.013837 | 6 |
| GO:1905710 | positive regulation of membrane permeability | 5/193 | 80/21081 | 0.000842 | 0.016331 | 0.013837 | 5 |
| GO:0001959 | regulation of cytokine-mediated signaling pathway | 8/193 | 216/21081 | 0.000872 | 0.016522 | 0.013999 | 8 |
| GO:0006109 | regulation of carbohydrate metabolic process | 8/193 | 216/21081 | 0.000872 | 0.016522 | 0.013999 | 8 |
| GO:0030308 | negative regulation of cell growth | 8/193 | 216/21081 | 0.000872 | 0.016522 | 0.013999 | 8 |
| GO:1900542 | regulation of purine nucleotide metabolic process | 6/193 | 121/21081 | 0.000876 | 0.016522 | 0.013999 | 6 |
| GO:1903311 | regulation of mRNA metabolic process | 11/193 | 386/21081 | 0.000897 | 0.01681 | 0.014242 | 11 |
| GO:0090092 | regulation of transmembrane receptor protein serine/threonine kinase signaling pathway | 9/193 | 272/21081 | 0.000936 | 0.017403 | 0.014745 | 9 |
| GO:0090287 | regulation of cellular response to growth factor stimulus | 10/193 | 329/21081 | 0.000943 | 0.017403 | 0.014745 | 10 |
| GO:0006140 | regulation of nucleotide metabolic process | 6/193 | 123/21081 | 0.000954 | 0.017403 | 0.014745 | 6 |
| GO:2001237 | negative regulation of extrinsic apoptotic signaling pathway | 6/193 | 123/21081 | 0.000954 | 0.017403 | 0.014745 | 6 |
| GO:0060716 | labyrinthine layer blood vessel development | 3/193 | 22/21081 | 0.001023 | 0.018341 | 0.01554 | 3 |
| GO:0034620 | cellular response to unfolded protein | 7/193 | 171/21081 | 0.001025 | 0.018341 | 0.01554 | 7 |
| GO:1902600 | proton transmembrane transport | 7/193 | 171/21081 | 0.001025 | 0.018341 | 0.01554 | 7 |
| GO:0009409 | response to cold | 4/193 | 49/21081 | 0.001046 | 0.018589 | 0.015749 | 4 |
| GO:0055067 | monovalent inorganic cation homeostasis | 7/193 | 172/21081 | 0.001061 | 0.018732 | 0.015871 | 7 |
| GO:1903578 | regulation of ATP metabolic process | 6/193 | 126/21081 | 0.001081 | 0.018972 | 0.016075 | 6 |
| GO:0007088 | regulation of mitotic nuclear division | 7/193 | 174/21081 | 0.001134 | 0.01978 | 0.016759 | 7 |
| GO:0046031 | ADP metabolic process | 6/193 | 128/21081 | 0.001173 | 0.020324 | 0.01722 | 6 |
| GO:0002753 | cytoplasmic pattern recognition receptor signaling pathway | 5/193 | 87/21081 | 0.00123 | 0.021177 | 0.017943 | 5 |
| GO:0034612 | response to tumor necrosis factor | 11/193 | 403/21081 | 0.001269 | 0.021717 | 0.0184 | 11 |
| GO:0060759 | regulation of response to cytokine stimulus | 8/193 | 230/21081 | 0.001304 | 0.022181 | 0.018793 | 8 |
| GO:0034599 | cellular response to oxidative stress | 10/193 | 344/21081 | 0.001318 | 0.022278 | 0.018875 | 10 |
| GO:0006896 | Golgi to vacuole transport | 3/193 | 24/21081 | 0.001327 | 0.022302 | 0.018896 | 3 |
| GO:0009416 | response to light stimulus | 10/193 | 345/21081 | 0.001346 | 0.02249 | 0.019055 | 10 |
| GO:0046902 | regulation of mitochondrial membrane permeability | 5/193 | 89/21081 | 0.001361 | 0.0226 | 0.019148 | 5 |
| GO:2001236 | regulation of extrinsic apoptotic signaling pathway | 7/193 | 181/21081 | 0.001424 | 0.023411 | 0.019836 | 7 |
| GO:1902903 | regulation of supramolecular fiber organization | 11/193 | 409/21081 | 0.001427 | 0.023411 | 0.019836 | 11 |
| GO:0038034 | signal transduction in absence of ligand | 5/193 | 91/21081 | 0.001503 | 0.024368 | 0.020646 | 5 |
| GO:0097192 | extrinsic apoptotic signaling pathway in absence of ligand | 5/193 | 91/21081 | 0.001503 | 0.024368 | 0.020646 | 5 |
| GO:0002262 | myeloid cell homeostasis | 7/193 | 183/21081 | 0.001517 | 0.024419 | 0.02069 | 7 |
| GO:0019058 | viral life cycle | 11/193 | 413/21081 | 0.001541 | 0.024419 | 0.02069 | 11 |
| GO:0007052 | mitotic spindle organization | 6/193 | 135/21081 | 0.001541 | 0.024419 | 0.02069 | 6 |
| GO:0030101 | natural killer cell activation | 6/193 | 135/21081 | 0.001541 | 0.024419 | 0.02069 | 6 |
| GO:0001913 | T cell mediated cytotoxicity | 5/193 | 92/21081 | 0.001577 | 0.024853 | 0.021057 | 5 |
| GO:0006165 | nucleoside diphosphate phosphorylation | 6/193 | 136/21081 | 0.0016 | 0.024999 | 0.021181 | 6 |
| GO:0014888 | striated muscle adaptation | 4/193 | 55/21081 | 0.001613 | 0.024999 | 0.021181 | 4 |
| GO:0016482 | cytosolic transport | 7/193 | 185/21081 | 0.001614 | 0.024999 | 0.021181 | 7 |
| GO:0016125 | sterol metabolic process | 7/193 | 187/21081 | 0.001716 | 0.026259 | 0.022248 | 7 |
| GO:0043500 | muscle adaptation | 6/193 | 138/21081 | 0.001723 | 0.026259 | 0.022248 | 6 |
| GO:0046939 | nucleotide phosphorylation | 6/193 | 138/21081 | 0.001723 | 0.026259 | 0.022248 | 6 |
| GO:0035966 | response to topologically incorrect protein | 8/193 | 242/21081 | 0.001797 | 0.027224 | 0.023066 | 8 |
| GO:0045953 | negative regulation of natural killer cell mediated cytotoxicity | 5/193 | 95/21081 | 0.001818 | 0.027399 | 0.023215 | 5 |
| GO:0009135 | purine nucleoside diphosphate metabolic process | 6/193 | 140/21081 | 0.001854 | 0.027638 | 0.023417 | 6 |
| GO:0009179 | purine ribonucleoside diphosphate metabolic process | 6/193 | 140/21081 | 0.001854 | 0.027638 | 0.023417 | 6 |
| GO:0002716 | negative regulation of natural killer cell mediated immunity | 5/193 | 96/21081 | 0.001904 | 0.028083 | 0.023794 | 5 |
| GO:0031397 | negative regulation of protein ubiquitination | 5/193 | 96/21081 | 0.001904 | 0.028083 | 0.023794 | 5 |
| GO:0090101 | negative regulation of transmembrane receptor protein serine/threonine kinase signaling pathway | 6/193 | 141/21081 | 0.001922 | 0.028196 | 0.02389 | 6 |
| GO:0090288 | negative regulation of cellular response to growth factor stimulus | 7/193 | 191/21081 | 0.001935 | 0.028236 | 0.023924 | 7 |
| GO:0044262 | cellular carbohydrate metabolic process | 9/193 | 303/21081 | 0.00197 | 0.028603 | 0.024234 | 9 |
| GO:0009185 | ribonucleoside diphosphate metabolic process | 6/193 | 143/21081 | 0.002063 | 0.029505 | 0.024999 | 6 |
| GO:0042269 | regulation of natural killer cell mediated cytotoxicity | 6/193 | 143/21081 | 0.002063 | 0.029505 | 0.024999 | 6 |
| GO:0062012 | regulation of small molecule metabolic process | 12/193 | 494/21081 | 0.002064 | 0.029505 | 0.024999 | 12 |
| GO:0032436 | positive regulation of proteasomal ubiquitin-dependent protein catabolic process | 5/193 | 99/21081 | 0.002179 | 0.030834 | 0.026125 | 5 |
| GO:0097194 | execution phase of apoptosis | 5/193 | 99/21081 | 0.002179 | 0.030834 | 0.026125 | 5 |
| GO:0050732 | negative regulation of peptidyl-tyrosine phosphorylation | 4/193 | 60/21081 | 0.002226 | 0.031333 | 0.026547 | 4 |
| GO:0001911 | negative regulation of leukocyte mediated cytotoxicity | 5/193 | 100/21081 | 0.002277 | 0.031895 | 0.027023 | 5 |
| GO:0071356 | cellular response to tumor necrosis factor | 10/193 | 372/21081 | 0.002338 | 0.032586 | 0.027609 | 10 |
| GO:0048008 | platelet-derived growth factor receptor signaling pathway | 4/193 | 61/21081 | 0.002365 | 0.032651 | 0.027664 | 4 |
| GO:0002715 | regulation of natural killer cell mediated immunity | 6/193 | 147/21081 | 0.00237 | 0.032651 | 0.027664 | 6 |
| GO:0090559 | regulation of membrane permeability | 5/193 | 101/21081 | 0.002378 | 0.032651 | 0.027664 | 5 |
| GO:0071478 | cellular response to radiation | 7/193 | 200/21081 | 0.002506 | 0.034239 | 0.029009 | 7 |
| GO:0051783 | regulation of nuclear division | 7/193 | 201/21081 | 0.002577 | 0.035033 | 0.029682 | 7 |
| GO:0051495 | positive regulation of cytoskeleton organization | 8/193 | 258/21081 | 0.002671 | 0.036126 | 0.030608 | 8 |
| GO:0031342 | negative regulation of cell killing | 5/193 | 104/21081 | 0.002701 | 0.036365 | 0.030811 | 5 |
| GO:0035967 | cellular response to topologically incorrect protein | 7/193 | 204/21081 | 0.002799 | 0.037487 | 0.031761 | 7 |
| GO:1904837 | beta-catenin-TCF complex assembly | 3/193 | 31/21081 | 0.002812 | 0.037487 | 0.031761 | 3 |
| GO:0051092 | positive regulation of NF-kappaB transcription factor activity | 7/193 | 205/21081 | 0.002876 | 0.038157 | 0.032329 | 7 |
| GO:0045444 | fat cell differentiation | 8/193 | 263/21081 | 0.003003 | 0.039652 | 0.033596 | 8 |
| GO:0007265 | Ras protein signal transduction | 10/193 | 386/21081 | 0.003047 | 0.040044 | 0.033928 | 10 |
| GO:0018200 | peptidyl-glutamic acid modification | 3/193 | 32/21081 | 0.003082 | 0.040313 | 0.034156 | 3 |
| GO:2000272 | negative regulation of signaling receptor activity | 4/193 | 66/21081 | 0.003153 | 0.040825 | 0.03459 | 4 |
| GO:1903321 | negative regulation of protein modification by small protein conjugation or removal | 5/193 | 108/21081 | 0.00318 | 0.040825 | 0.03459 | 5 |
| GO:1905897 | regulation of response to endoplasmic reticulum stress | 5/193 | 108/21081 | 0.00318 | 0.040825 | 0.03459 | 5 |
| GO:2001243 | negative regulation of intrinsic apoptotic signaling pathway | 5/193 | 108/21081 | 0.00318 | 0.040825 | 0.03459 | 5 |
| GO:0006402 | mRNA catabolic process | 11/193 | 454/21081 | 0.003203 | 0.040924 | 0.034674 | 11 |
| GO:0007006 | mitochondrial membrane organization | 6/193 | 157/21081 | 0.003286 | 0.041413 | 0.035088 | 6 |
| GO:0106106 | cold-induced thermogenesis | 6/193 | 157/21081 | 0.003286 | 0.041413 | 0.035088 | 6 |
| GO:0120161 | regulation of cold-induced thermogenesis | 6/193 | 157/21081 | 0.003286 | 0.041413 | 0.035088 | 6 |
| GO:0046320 | regulation of fatty acid oxidation | 3/193 | 33/21081 | 0.003368 | 0.042153 | 0.035714 | 3 |
| GO:0006090 | pyruvate metabolic process | 6/193 | 158/21081 | 0.00339 | 0.042153 | 0.035714 | 6 |
| GO:0009132 | nucleoside diphosphate metabolic process | 6/193 | 158/21081 | 0.00339 | 0.042153 | 0.035714 | 6 |
| GO:0090160 | Golgi to lysosome transport | 2/193 | 10/21081 | 0.003575 | 0.044085 | 0.037352 | 2 |
| GO:0019395 | fatty acid oxidation | 5/193 | 111/21081 | 0.003577 | 0.044085 | 0.037352 | 5 |
| GO:0062197 | cellular response to chemical stress | 10/193 | 396/21081 | 0.003651 | 0.044795 | 0.037953 | 10 |
| GO:0032677 | regulation of interleukin-8 production | 5/193 | 112/21081 | 0.003717 | 0.045405 | 0.03847 | 5 |
| GO:0010657 | muscle cell apoptotic process | 5/193 | 113/21081 | 0.003861 | 0.046955 | 0.039783 | 5 |
| GO:1902850 | microtubule cytoskeleton organization involved in mitosis | 6/193 | 163/21081 | 0.003949 | 0.047813 | 0.040511 | 6 |
