# Supplementary material for: Exploring the Pleiotropic Genes and Therapeutic Targets Associated with Heart Failure and Chronic Kidney Disease by Integrating metaCCA and SGLT2 Inhibitors' Target Prediction
Source: Biomed Res Int. 2021 Sep 8;2021:4229194. doi: 10.1155/2021/4229194 (PMC8443964; doi:10.1155/2021/4229194)
Supplement: Supplementary 5 — Table S5: potential genes KEGG. [file 4229194.f5.docx]

| ID | Description | GeneRatio | BgRatio | pvalue | p.adjust | qvalue | Count |
| --- | --- | --- | --- | --- | --- | --- | --- |
| hsa05215 | Prostate cancer | 7/96 | 97/8105 | 0.000141 | 0.023402 | 0.020727 | 7 |
| hsa04910 | Insulin signaling pathway | 8/96 | 137/8105 | 0.000206 | 0.023402 | 0.020727 | 8 |
| hsa05222 | Small cell lung cancer | 6/96 | 92/8105 | 0.000741 | 0.056093 | 0.049682 | 6 |
| hsa04920 | Adipocytokine signaling pathway | 5/96 | 69/8105 | 0.001297 | 0.073598 | 0.065185 | 5 |
| hsa04922 | Glucagon signaling pathway | 6/96 | 107/8105 | 0.00163 | 0.074007 | 0.065547 | 6 |
| hsa05145 | Toxoplasmosis | 6/96 | 112/8105 | 0.002058 | 0.077871 | 0.06897 | 6 |
| hsa04152 | AMPK signaling pathway | 6/96 | 120/8105 | 0.002914 | 0.084564 | 0.074898 | 6 |
| hsa04151 | PI3K-Akt signaling pathway | 11/96 | 354/8105 | 0.00298 | 0.084564 | 0.074898 | 11 |
| hsa04068 | FoxO signaling pathway | 6/96 | 131/8105 | 0.004494 | 0.102587 | 0.090861 | 6 |
| hsa04923 | Regulation of lipolysis in adipocytes | 4/96 | 57/8105 | 0.004519 | 0.102587 | 0.090861 | 4 |
| hsa04371 | Apelin signaling pathway | 6/96 | 137/8105 | 0.005585 | 0.106554 | 0.094374 | 6 |
| hsa04014 | Ras signaling pathway | 8/96 | 232/8105 | 0.006079 | 0.106554 | 0.094374 | 8 |
| hsa04213 | Longevity regulating pathway - multiple species | 4/96 | 62/8105 | 0.006102 | 0.106554 | 0.094374 | 4 |
| hsa05167 | Kaposi sarcoma-associated herpesvirus infection | 7/96 | 193/8105 | 0.007789 | 0.12629 | 0.111854 | 7 |
| hsa04931 | Insulin resistance | 5/96 | 108/8105 | 0.008966 | 0.128815 | 0.114091 | 5 |
| hsa05120 | Epithelial cell signaling in Helicobacter pylori infection | 4/96 | 70/8105 | 0.009337 | 0.128815 | 0.114091 | 4 |
| hsa04520 | Adherens junction | 4/96 | 71/8105 | 0.009806 | 0.128815 | 0.114091 | 4 |
| hsa05218 | Melanoma | 4/96 | 72/8105 | 0.01029 | 0.128815 | 0.114091 | 4 |
| hsa04725 | Cholinergic synapse | 5/96 | 113/8105 | 0.010782 | 0.128815 | 0.114091 | 5 |
| hsa05220 | Chronic myeloid leukemia | 4/96 | 76/8105 | 0.012383 | 0.140544 | 0.124479 | 4 |
| hsa05417 | Lipid and atherosclerosis | 7/96 | 215/8105 | 0.013606 | 0.145679 | 0.129027 | 7 |
| hsa01521 | EGFR tyrosine kinase inhibitor resistance | 4/96 | 79/8105 | 0.014119 | 0.145679 | 0.129027 | 4 |
| hsa05165 | Human papillomavirus infection | 9/96 | 331/8105 | 0.016211 | 0.159997 | 0.141708 | 9 |
| hsa04142 | Lysosome | 5/96 | 128/8105 | 0.017707 | 0.167476 | 0.148333 | 5 |
| hsa00511 | Other glycan degradation | 2/96 | 18/8105 | 0.018778 | 0.170503 | 0.151014 | 2 |
| hsa04211 | Longevity regulating pathway | 4/96 | 89/8105 | 0.020991 | 0.177623 | 0.15732 | 4 |
| hsa04979 | Cholesterol metabolism | 3/96 | 50/8105 | 0.021127 | 0.177623 | 0.15732 | 3 |
| hsa05132 | Salmonella infection | 7/96 | 249/8105 | 0.027932 | 0.223975 | 0.198374 | 7 |
| hsa04914 | Progesterone-mediated oocyte maturation | 4/96 | 100/8105 | 0.03058 | 0.223975 | 0.198374 | 4 |
| hsa04933 | AGE-RAGE signaling pathway in diabetic complications | 4/96 | 100/8105 | 0.03058 | 0.223975 | 0.198374 | 4 |
| hsa05226 | Gastric cancer | 5/96 | 149/8105 | 0.031589 | 0.223975 | 0.198374 | 5 |
| hsa04510 | Focal adhesion | 6/96 | 201/8105 | 0.03171 | 0.223975 | 0.198374 | 6 |
| hsa05142 | Chagas disease | 4/96 | 102/8105 | 0.03256 | 0.223975 | 0.198374 | 4 |
| hsa04668 | TNF signaling pathway | 4/96 | 112/8105 | 0.043571 | 0.290344 | 0.257156 | 4 |
| hsa05221 | Acute myeloid leukemia | 3/96 | 67/8105 | 0.044767 | 0.290344 | 0.257156 | 3 |
| hsa05211 | Renal cell carcinoma | 3/96 | 69/8105 | 0.048151 | 0.303616 | 0.268911 | 3 |
| hsa04071 | Sphingolipid signaling pathway | 4/96 | 119/8105 | 0.052384 | 0.321381 | 0.284645 | 4 |
| hsa04010 | MAPK signaling pathway | 7/96 | 294/8105 | 0.059273 | 0.354079 | 0.313605 | 7 |
| hsa04971 | Gastric acid secretion | 3/96 | 76/8105 | 0.060947 | 0.354742 | 0.314193 | 3 |
| hsa05216 | Thyroid cancer | 2/96 | 37/8105 | 0.070766 | 0.401595 | 0.35569 | 2 |
| hsa04210 | Apoptosis | 4/96 | 136/8105 | 0.077517 | 0.416437 | 0.368836 | 4 |
| hsa04144 | Endocytosis | 6/96 | 252/8105 | 0.078013 | 0.416437 | 0.368836 | 6 |
| hsa04915 | Estrogen signaling pathway | 4/96 | 138/8105 | 0.080812 | 0.416437 | 0.368836 | 4 |
| hsa04742 | Taste transduction | 3/96 | 86/8105 | 0.081668 | 0.416437 | 0.368836 | 3 |
| hsa04512 | ECM-receptor interaction | 3/96 | 88/8105 | 0.086136 | 0.416437 | 0.368836 | 3 |
| hsa05235 | PD-L1 expression and PD-1 checkpoint pathway in cancer | 3/96 | 89/8105 | 0.088408 | 0.416437 | 0.368836 | 3 |
| hsa05017 | Spinocerebellar ataxia | 4/96 | 143/8105 | 0.089351 | 0.416437 | 0.368836 | 4 |
| hsa05032 | Morphine addiction | 3/96 | 91/8105 | 0.093029 | 0.416437 | 0.368836 | 3 |
| hsa05224 | Breast cancer | 4/96 | 147/8105 | 0.096483 | 0.416437 | 0.368836 | 4 |
| hsa04072 | Phospholipase D signaling pathway | 4/96 | 148/8105 | 0.098308 | 0.416437 | 0.368836 | 4 |
| hsa04350 | TGF-beta signaling pathway | 3/96 | 94/8105 | 0.100144 | 0.416437 | 0.368836 | 3 |
| hsa04932 | Non-alcoholic fatty liver disease | 4/96 | 150/8105 | 0.102005 | 0.416437 | 0.368836 | 4 |
| hsa05170 | Human immunodeficiency virus 1 infection | 5/96 | 212/8105 | 0.106422 | 0.416437 | 0.368836 | 5 |
| hsa04973 | Carbohydrate digestion and absorption | 2/96 | 47/8105 | 0.106582 | 0.416437 | 0.368836 | 2 |
| hsa04666 | Fc gamma R-mediated phagocytosis | 3/96 | 97/8105 | 0.107471 | 0.416437 | 0.368836 | 3 |
| hsa05231 | Choline metabolism in cancer | 3/96 | 98/8105 | 0.10996 | 0.416437 | 0.368836 | 3 |
| hsa04150 | mTOR signaling pathway | 4/96 | 155/8105 | 0.111525 | 0.416437 | 0.368836 | 4 |
| hsa00072 | Synthesis and degradation of ketone bodies | 1/96 | 10/8105 | 0.112387 | 0.416437 | 0.368836 | 1 |
| hsa04024 | cAMP signaling pathway | 5/96 | 216/8105 | 0.112846 | 0.416437 | 0.368836 | 5 |
| hsa04218 | Cellular senescence | 4/96 | 156/8105 | 0.113476 | 0.416437 | 0.368836 | 4 |
| hsa04810 | Regulation of actin cytoskeleton | 5/96 | 218/8105 | 0.116127 | 0.416437 | 0.368836 | 5 |
| hsa05166 | Human T-cell leukemia virus 1 infection | 5/96 | 219/8105 | 0.117785 | 0.416437 | 0.368836 | 5 |
| hsa05110 | Vibrio cholerae infection | 2/96 | 50/8105 | 0.118116 | 0.416437 | 0.368836 | 2 |
| hsa04217 | Necroptosis | 4/96 | 159/8105 | 0.119419 | 0.416437 | 0.368836 | 4 |
| hsa05146 | Amoebiasis | 3/96 | 102/8105 | 0.120133 | 0.416437 | 0.368836 | 3 |
| hsa04974 | Protein digestion and absorption | 3/96 | 103/8105 | 0.12273 | 0.416437 | 0.368836 | 3 |
| hsa00430 | Taurine and hypotaurine metabolism | 1/96 | 11/8105 | 0.122913 | 0.416437 | 0.368836 | 1 |
| hsa05161 | Hepatitis B | 4/96 | 162/8105 | 0.125495 | 0.418931 | 0.371045 | 4 |
| hsa04659 | Th17 cell differentiation | 3/96 | 107/8105 | 0.133319 | 0.438602 | 0.388467 | 3 |
| hsa04022 | cGMP-PKG signaling pathway | 4/96 | 167/8105 | 0.135908 | 0.440731 | 0.390353 | 4 |
| hsa04066 | HIF-1 signaling pathway | 3/96 | 109/8105 | 0.138731 | 0.443549 | 0.392849 | 3 |
| hsa04141 | Protein processing in endoplasmic reticulum | 4/96 | 171/8105 | 0.144487 | 0.449903 | 0.398476 | 4 |
| hsa05134 | Legionellosis | 2/96 | 57/8105 | 0.146125 | 0.449903 | 0.398476 | 2 |
| hsa05164 | Influenza A | 4/96 | 172/8105 | 0.146664 | 0.449903 | 0.398476 | 4 |
| hsa05213 | Endometrial cancer | 2/96 | 58/8105 | 0.150234 | 0.454708 | 0.402732 | 2 |
| hsa00604 | Glycosphingolipid biosynthesis - ganglio series | 1/96 | 15/8105 | 0.163797 | 0.485577 | 0.430073 | 1 |
| hsa04722 | Neurotrophin signaling pathway | 3/96 | 119/8105 | 0.16685 | 0.485577 | 0.430073 | 3 |
| hsa04935 | Growth hormone synthesis, secretion and action | 3/96 | 119/8105 | 0.16685 | 0.485577 | 0.430073 | 3 |
| hsa04919 | Thyroid hormone signaling pathway | 3/96 | 121/8105 | 0.172667 | 0.496144 | 0.439432 | 3 |
| hsa04110 | Cell cycle | 3/96 | 124/8105 | 0.181499 | 0.515004 | 0.456136 | 3 |
| hsa04664 | Fc epsilon RI signaling pathway | 2/96 | 68/8105 | 0.192419 | 0.516828 | 0.457752 | 2 |
| hsa04062 | Chemokine signaling pathway | 4/96 | 192/8105 | 0.192731 | 0.516828 | 0.457752 | 4 |
| hsa05202 | Transcriptional misregulation in cancer | 4/96 | 192/8105 | 0.192731 | 0.516828 | 0.457752 | 4 |
| hsa04380 | Osteoclast differentiation | 3/96 | 128/8105 | 0.193463 | 0.516828 | 0.457752 | 3 |
| hsa04926 | Relaxin signaling pathway | 3/96 | 129/8105 | 0.196485 | 0.516828 | 0.457752 | 3 |
| hsa00230 | Purine metabolism | 3/96 | 130/8105 | 0.199519 | 0.516828 | 0.457752 | 3 |
| hsa04917 | Prolactin signaling pathway | 2/96 | 70/8105 | 0.201044 | 0.516828 | 0.457752 | 2 |
| hsa05230 | Central carbon metabolism in cancer | 2/96 | 70/8105 | 0.201044 | 0.516828 | 0.457752 | 2 |
| hsa00531 | Glycosaminoglycan degradation | 1/96 | 19/8105 | 0.202795 | 0.516828 | 0.457752 | 1 |
| hsa05130 | Pathogenic Escherichia coli infection | 4/96 | 197/8105 | 0.20491 | 0.516828 | 0.457752 | 4 |
| hsa05223 | Non-small cell lung cancer | 2/96 | 72/8105 | 0.209715 | 0.520405 | 0.46092 | 2 |
| hsa00100 | Steroid biosynthesis | 1/96 | 20/8105 | 0.212259 | 0.520405 | 0.46092 | 1 |
| hsa04080 | Neuroactive ligand-receptor interaction | 6/96 | 341/8105 | 0.216716 | 0.520405 | 0.46092 | 6 |
| hsa00770 | Pantothenate and CoA biosynthesis | 1/96 | 21/8105 | 0.221613 | 0.520405 | 0.46092 | 1 |
| hsa05203 | Viral carcinogenesis | 4/96 | 204/8105 | 0.222331 | 0.520405 | 0.46092 | 4 |
| hsa04918 | Thyroid hormone synthesis | 2/96 | 75/8105 | 0.222791 | 0.520405 | 0.46092 | 2 |
| hsa05212 | Pancreatic cancer | 2/96 | 76/8105 | 0.227165 | 0.520405 | 0.46092 | 2 |
| hsa05162 | Measles | 3/96 | 139/8105 | 0.227301 | 0.520405 | 0.46092 | 3 |
| hsa05418 | Fluid shear stress and atherosclerosis | 3/96 | 139/8105 | 0.227301 | 0.520405 | 0.46092 | 3 |
| hsa04120 | Ubiquitin mediated proteolysis | 3/96 | 140/8105 | 0.230435 | 0.520405 | 0.46092 | 3 |
| hsa05100 | Bacterial invasion of epithelial cells | 2/96 | 77/8105 | 0.231546 | 0.520405 | 0.46092 | 2 |
| hsa04612 | Antigen processing and presentation | 2/96 | 78/8105 | 0.235932 | 0.523577 | 0.463729 | 2 |
| hsa04015 | Rap1 signaling pathway | 4/96 | 210/8105 | 0.23757 | 0.523577 | 0.463729 | 4 |
| hsa04550 | Signaling pathways regulating pluripotency of stem cells | 3/96 | 143/8105 | 0.239885 | 0.523596 | 0.463746 | 3 |
| hsa00534 | Glycosaminoglycan biosynthesis - heparan sulfate / heparin | 1/96 | 24/8105 | 0.249019 | 0.537841 | 0.476362 | 1 |
| hsa04146 | Peroxisome | 2/96 | 82/8105 | 0.25352 | 0.537841 | 0.476362 | 2 |
| hsa04662 | B cell receptor signaling pathway | 2/96 | 82/8105 | 0.25352 | 0.537841 | 0.476362 | 2 |
| hsa04012 | ErbB signaling pathway | 2/96 | 85/8105 | 0.266735 | 0.55554 | 0.492038 | 2 |
| hsa00563 | Glycosylphosphatidylinositol (GPI)-anchor biosynthesis | 1/96 | 26/8105 | 0.266757 | 0.55554 | 0.492038 | 1 |
| hsa05210 | Colorectal cancer | 2/96 | 86/8105 | 0.271141 | 0.556101 | 0.492535 | 2 |
| hsa05010 | Alzheimer disease | 6/96 | 369/8105 | 0.271926 | 0.556101 | 0.492535 | 6 |
| hsa04966 | Collecting duct acid secretion | 1/96 | 27/8105 | 0.27547 | 0.557246 | 0.49355 | 1 |
| hsa00650 | Butanoate metabolism | 1/96 | 28/8105 | 0.28408 | 0.557246 | 0.49355 | 1 |
| hsa04727 | GABAergic synapse | 2/96 | 89/8105 | 0.284355 | 0.557246 | 0.49355 | 2 |
| hsa05160 | Hepatitis C | 3/96 | 157/8105 | 0.284741 | 0.557246 | 0.49355 | 3 |
| hsa04976 | Bile secretion | 2/96 | 90/8105 | 0.288756 | 0.557246 | 0.49355 | 2 |
| hsa00591 | Linoleic acid metabolism | 1/96 | 29/8105 | 0.292589 | 0.557246 | 0.49355 | 1 |
| hsa04744 | Phototransduction | 1/96 | 29/8105 | 0.292589 | 0.557246 | 0.49355 | 1 |
| hsa04658 | Th1 and Th2 cell differentiation | 2/96 | 92/8105 | 0.29755 | 0.557246 | 0.49355 | 2 |
| hsa04630 | JAK-STAT signaling pathway | 3/96 | 162/8105 | 0.300961 | 0.557246 | 0.49355 | 3 |
| hsa00410 | beta-Alanine metabolism | 1/96 | 30/8105 | 0.300998 | 0.557246 | 0.49355 | 1 |
| hsa05206 | MicroRNAs in cancer | 5/96 | 310/8105 | 0.305641 | 0.557246 | 0.49355 | 5 |
| hsa04657 | IL-17 signaling pathway | 2/96 | 94/8105 | 0.306328 | 0.557246 | 0.49355 | 2 |
| hsa00052 | Galactose metabolism | 1/96 | 31/8105 | 0.309309 | 0.557246 | 0.49355 | 1 |
| hsa01523 | Antifolate resistance | 1/96 | 31/8105 | 0.309309 | 0.557246 | 0.49355 | 1 |
| hsa04710 | Circadian rhythm | 1/96 | 31/8105 | 0.309309 | 0.557246 | 0.49355 | 1 |
| hsa04020 | Calcium signaling pathway | 4/96 | 240/8105 | 0.316769 | 0.559996 | 0.495986 | 4 |
| hsa04215 | Apoptosis - multiple species | 1/96 | 32/8105 | 0.317521 | 0.559996 | 0.495986 | 1 |
| hsa05225 | Hepatocellular carcinoma | 3/96 | 168/8105 | 0.320493 | 0.559996 | 0.495986 | 3 |
| hsa04925 | Aldosterone synthesis and secretion | 2/96 | 98/8105 | 0.323822 | 0.559996 | 0.495986 | 2 |
| hsa00051 | Fructose and mannose metabolism | 1/96 | 33/8105 | 0.325637 | 0.559996 | 0.495986 | 1 |
| hsa04130 | SNARE interactions in vesicular transport | 1/96 | 33/8105 | 0.325637 | 0.559996 | 0.495986 | 1 |
| hsa05131 | Shigellosis | 4/96 | 246/8105 | 0.332975 | 0.568311 | 0.50335 | 4 |
| hsa04916 | Melanogenesis | 2/96 | 101/8105 | 0.336873 | 0.570674 | 0.505442 | 2 |
| hsa04972 | Pancreatic secretion | 2/96 | 102/8105 | 0.341208 | 0.571341 | 0.506033 | 2 |
| hsa04064 | NF-kappa B signaling pathway | 2/96 | 104/8105 | 0.349852 | 0.571341 | 0.506033 | 2 |
| hsa04620 | Toll-like receptor signaling pathway | 2/96 | 104/8105 | 0.349852 | 0.571341 | 0.506033 | 2 |
| hsa04625 | C-type lectin receptor signaling pathway | 2/96 | 104/8105 | 0.349852 | 0.571341 | 0.506033 | 2 |
| hsa04660 | T cell receptor signaling pathway | 2/96 | 104/8105 | 0.349852 | 0.571341 | 0.506033 | 2 |
| hsa05143 | African trypanosomiasis | 1/96 | 37/8105 | 0.357155 | 0.578888 | 0.512718 | 1 |
| hsa05152 | Tuberculosis | 3/96 | 180/8105 | 0.359574 | 0.578888 | 0.512718 | 3 |
| hsa04360 | Axon guidance | 3/96 | 182/8105 | 0.366069 | 0.585194 | 0.518303 | 3 |
| hsa05168 | Herpes simplex virus 1 infection | 7/96 | 498/8105 | 0.37667 | 0.594636 | 0.526666 | 7 |
| hsa00260 | Glycine, serine and threonine metabolism | 1/96 | 40/8105 | 0.379833 | 0.594636 | 0.526666 | 1 |
| hsa05033 | Nicotine addiction | 1/96 | 40/8105 | 0.379833 | 0.594636 | 0.526666 | 1 |
| hsa04724 | Glutamatergic synapse | 2/96 | 114/8105 | 0.392454 | 0.610186 | 0.540438 | 2 |
| hsa04726 | Serotonergic synapse | 2/96 | 115/8105 | 0.39665 | 0.612514 | 0.5425 | 2 |
| hsa03022 | Basal transcription factors | 1/96 | 45/8105 | 0.415884 | 0.637876 | 0.564963 | 1 |
| hsa03050 | Proteasome | 1/96 | 46/8105 | 0.422841 | 0.6399 | 0.566756 | 1 |
| hsa04930 | Type II diabetes mellitus | 1/96 | 46/8105 | 0.422841 | 0.6399 | 0.566756 | 1 |
| hsa03420 | Nucleotide excision repair | 1/96 | 47/8105 | 0.429716 | 0.642511 | 0.569068 | 1 |
| hsa05169 | Epstein-Barr virus infection | 3/96 | 202/8105 | 0.430228 | 0.642511 | 0.569068 | 3 |
| hsa00280 | Valine, leucine and isoleucine degradation | 1/96 | 48/8105 | 0.436511 | 0.647633 | 0.573605 | 1 |
| hsa05205 | Proteoglycans in cancer | 3/96 | 205/8105 | 0.439678 | 0.648096 | 0.574015 | 3 |
| hsa00600 | Sphingolipid metabolism | 1/96 | 49/8105 | 0.443225 | 0.649109 | 0.574913 | 1 |
| hsa00270 | Cysteine and methionine metabolism | 1/96 | 50/8105 | 0.449859 | 0.650434 | 0.576085 | 1 |
| hsa05144 | Malaria | 1/96 | 50/8105 | 0.449859 | 0.650434 | 0.576085 | 1 |
| hsa00330 | Arginine and proline metabolism | 1/96 | 51/8105 | 0.456416 | 0.655737 | 0.580783 | 1 |
| hsa04728 | Dopaminergic synapse | 2/96 | 132/8105 | 0.465783 | 0.664986 | 0.588974 | 2 |
| hsa05320 | Autoimmune thyroid disease | 1/96 | 53/8105 | 0.469298 | 0.665817 | 0.58971 | 1 |
| hsa03460 | Fanconi anemia pathway | 1/96 | 54/8105 | 0.475625 | 0.670602 | 0.593949 | 1 |
| hsa04140 | Autophagy - animal | 2/96 | 137/8105 | 0.485238 | 0.675542 | 0.598324 | 2 |
| hsa05135 | Yersinia infection | 2/96 | 137/8105 | 0.485238 | 0.675542 | 0.598324 | 2 |
| hsa04340 | Hedgehog signaling pathway | 1/96 | 56/8105 | 0.488057 | 0.675542 | 0.598324 | 1 |
| hsa05016 | Huntington disease | 4/96 | 306/8105 | 0.49326 | 0.678606 | 0.601038 | 4 |
| hsa05163 | Human cytomegalovirus infection | 3/96 | 225/8105 | 0.501035 | 0.679871 | 0.602158 | 3 |
| hsa04330 | Notch signaling pathway | 1/96 | 59/8105 | 0.506159 | 0.679871 | 0.602158 | 1 |
| hsa04370 | VEGF signaling pathway | 1/96 | 59/8105 | 0.506159 | 0.679871 | 0.602158 | 1 |
| hsa04978 | Mineral absorption | 1/96 | 59/8105 | 0.506159 | 0.679871 | 0.602158 | 1 |
| hsa00561 | Glycerolipid metabolism | 1/96 | 61/8105 | 0.517874 | 0.685265 | 0.606935 | 1 |
| hsa00590 | Arachidonic acid metabolism | 1/96 | 61/8105 | 0.517874 | 0.685265 | 0.606935 | 1 |
| hsa04714 | Thermogenesis | 3/96 | 232/8105 | 0.521724 | 0.685265 | 0.606935 | 3 |
| hsa04723 | Retrograde endocannabinoid signaling | 2/96 | 148/8105 | 0.526482 | 0.685265 | 0.606935 | 2 |
| hsa04623 | Cytosolic DNA-sensing pathway | 1/96 | 63/8105 | 0.529314 | 0.685265 | 0.606935 | 1 |
| hsa05217 | Basal cell carcinoma | 1/96 | 63/8105 | 0.529314 | 0.685265 | 0.606935 | 1 |
| hsa04261 | Adrenergic signaling in cardiomyocytes | 2/96 | 150/8105 | 0.533742 | 0.685265 | 0.606935 | 2 |
| hsa04929 | GnRH secretion | 1/96 | 64/8105 | 0.534933 | 0.685265 | 0.606935 | 1 |
| hsa03040 | Spliceosome | 2/96 | 151/8105 | 0.537344 | 0.685265 | 0.606935 | 2 |
| hsa00970 | Aminoacyl-tRNA biosynthesis | 1/96 | 66/8105 | 0.545972 | 0.691739 | 0.61267 | 1 |
| hsa04720 | Long-term potentiation | 1/96 | 67/8105 | 0.551394 | 0.691739 | 0.61267 | 1 |
| hsa04934 | Cushing syndrome | 2/96 | 155/8105 | 0.551563 | 0.691739 | 0.61267 | 2 |
| hsa00830 | Retinol metabolism | 1/96 | 68/8105 | 0.556751 | 0.69441 | 0.615035 | 1 |
| hsa04924 | Renin secretion | 1/96 | 69/8105 | 0.562046 | 0.697183 | 0.617491 | 1 |
| hsa04622 | RIG-I-like receptor signaling pathway | 1/96 | 70/8105 | 0.567278 | 0.699848 | 0.619852 | 1 |
| hsa00982 | Drug metabolism - cytochrome P450 | 1/96 | 72/8105 | 0.577557 | 0.703465 | 0.623055 | 1 |
| hsa00562 | Inositol phosphate metabolism | 1/96 | 73/8105 | 0.582605 | 0.703465 | 0.623055 | 1 |
| hsa01524 | Platinum drug resistance | 1/96 | 73/8105 | 0.582605 | 0.703465 | 0.623055 | 1 |
| hsa04115 | p53 signaling pathway | 1/96 | 73/8105 | 0.582605 | 0.703465 | 0.623055 | 1 |
| hsa04310 | Wnt signaling pathway | 2/96 | 166/8105 | 0.589086 | 0.706571 | 0.625806 | 2 |
| hsa05214 | Glioma | 1/96 | 75/8105 | 0.592524 | 0.706571 | 0.625806 | 1 |
| hsa05133 | Pertussis | 1/96 | 76/8105 | 0.597395 | 0.706571 | 0.625806 | 1 |
| hsa05140 | Leishmaniasis | 1/96 | 77/8105 | 0.602209 | 0.706571 | 0.625806 | 1 |
| hsa05412 | Arrhythmogenic right ventricular cardiomyopathy | 1/96 | 77/8105 | 0.602209 | 0.706571 | 0.625806 | 1 |
| hsa00980 | Metabolism of xenobiotics by cytochrome P450 | 1/96 | 78/8105 | 0.606966 | 0.706571 | 0.625806 | 1 |
| hsa04721 | Synaptic vesicle cycle | 1/96 | 78/8105 | 0.606966 | 0.706571 | 0.625806 | 1 |
| hsa05204 | Chemical carcinogenesis | 1/96 | 83/8105 | 0.629919 | 0.727879 | 0.644678 | 1 |
| hsa05014 | Amyotrophic lateral sclerosis | 4/96 | 364/8105 | 0.631683 | 0.727879 | 0.644678 | 4 |
| hsa04621 | NOD-like receptor signaling pathway | 2/96 | 181/8105 | 0.636476 | 0.728595 | 0.645312 | 2 |
| hsa04610 | Complement and coagulation cascades | 1/96 | 85/8105 | 0.638724 | 0.728595 | 0.645312 | 1 |
| hsa04911 | Insulin secretion | 1/96 | 86/8105 | 0.643048 | 0.72986 | 0.646433 | 1 |
| hsa03013 | RNA transport | 2/96 | 186/8105 | 0.651306 | 0.732181 | 0.648489 | 2 |
| hsa04540 | Gap junction | 1/96 | 88/8105 | 0.651544 | 0.732181 | 0.648489 | 1 |
| hsa05410 | Hypertrophic cardiomyopathy | 1/96 | 90/8105 | 0.65984 | 0.737851 | 0.65351 | 1 |
| hsa05022 | Pathways of neurodegeneration - multiple diseases | 5/96 | 475/8105 | 0.670639 | 0.740415 | 0.655782 | 5 |
| hsa04970 | Salivary secretion | 1/96 | 93/8105 | 0.671919 | 0.740415 | 0.655782 | 1 |
| hsa05323 | Rheumatoid arthritis | 1/96 | 93/8105 | 0.671919 | 0.740415 | 0.655782 | 1 |
| hsa04070 | Phosphatidylinositol signaling system | 1/96 | 97/8105 | 0.687365 | 0.74352 | 0.658531 | 1 |
| hsa04713 | Circadian entrainment | 1/96 | 97/8105 | 0.687365 | 0.74352 | 0.658531 | 1 |
| hsa00564 | Glycerophospholipid metabolism | 1/96 | 98/8105 | 0.691113 | 0.74352 | 0.658531 | 1 |
| hsa01522 | Endocrine resistance | 1/96 | 98/8105 | 0.691113 | 0.74352 | 0.658531 | 1 |
| hsa04750 | Inflammatory mediator regulation of TRP channels | 1/96 | 98/8105 | 0.691113 | 0.74352 | 0.658531 | 1 |
| hsa04611 | Platelet activation | 1/96 | 124/8105 | 0.774376 | 0.829167 | 0.734388 | 1 |
| hsa04114 | Oocyte meiosis | 1/96 | 129/8105 | 0.787626 | 0.839395 | 0.743448 | 1 |
| hsa04650 | Natural killer cell mediated cytotoxicity | 1/96 | 131/8105 | 0.792708 | 0.840863 | 0.744748 | 1 |
| hsa00190 | Oxidative phosphorylation | 1/96 | 133/8105 | 0.79767 | 0.842191 | 0.745924 | 1 |
| hsa04145 | Phagosome | 1/96 | 152/8105 | 0.839314 | 0.87798 | 0.777622 | 1 |
| hsa05020 | Prion disease | 2/96 | 273/8105 | 0.839637 | 0.87798 | 0.777622 | 2 |
| hsa04921 | Oxytocin signaling pathway | 1/96 | 154/8105 | 0.84317 | 0.87798 | 0.777622 | 1 |
| hsa04390 | Hippo signaling pathway | 1/96 | 157/8105 | 0.848783 | 0.879789 | 0.779224 | 1 |
| hsa04530 | Tight junction | 1/96 | 169/8105 | 0.869315 | 0.896975 | 0.794446 | 1 |
| hsa05034 | Alcoholism | 1/96 | 187/8105 | 0.89505 | 0.919066 | 0.814011 | 1 |
| hsa04613 | Neutrophil extracellular trap formation | 1/96 | 190/8105 | 0.898822 | 0.919066 | 0.814011 | 1 |
| hsa05415 | Diabetic cardiomyopathy | 1/96 | 203/8105 | 0.913675 | 0.930064 | 0.823752 | 1 |
| hsa05171 | Coronavirus disease - COVID-19 | 1/96 | 232/8105 | 0.939478 | 0.95206 | 0.843234 | 1 |
| hsa05012 | Parkinson disease | 1/96 | 249/8105 | 0.950882 | 0.959334 | 0.849677 | 1 |
| hsa04060 | Cytokine-cytokine receptor interaction | 1/96 | 295/8105 | 0.972145 | 0.976446 | 0.864833 | 1 |
| hsa04740 | Olfactory transduction | 1/96 | 443/8105 | 0.995612 | 0.995612 | 0.881808 | 1 |
